# Nonmuscle myosin IIA (NMIIA) regulates anisotropic cell tension to maintain the hexagonal packing of mouse lens meridional row cells

**DOI:** 10.1091/mbc.E25-04-0154

**Published:** 2025-08-20

**Authors:** Sadia T. Islam, Yiwen Tang, Heather Boliver, Dapeng Bi, Velia M. Fowler

**Affiliations:** ^a^Department of Biological Sciences, University of Delaware, Newark, DE 19716; ^b^Department of Physics, Northeastern University, Boston, MA 02115; ^c^Center for Theoretical Biological Physics, Northeastern University, Boston, MA 02115; University of Queensland

## Abstract

The mouse ocular lens is an excellent vertebrate model for epithelial cell hexagonal packing during tissue morphogenesis. As lens epithelial cells differentiate into fiber cells, the epithelial cells rearrange into hexagonally packed meridional row (MR) cells that further differentiate to form fiber cells. We previously reported that the nonmuscle myosin IIA (NMIIA)-E1841K mutation, which alters NMIIA bipolar filament assembly, significantly disrupts MR cell hexagonal packing. Immunofluorescence microscopy of MR cells demonstrates increased enrichment of NMIIA, N-cadherin, and vinculin at anterior–posterior (AP)-oriented sides of control MR cells, but equal distributions on all sides of mutant MR cells. Furthermore, F-actin is uniformly distributed around all edges of control MR cells but reduced at the AP-oriented edges of mutant MR cells. Using Bayesian Mechanical Inference, we discovered that MR cells in control lenses exhibit anisotropic junctional tension, in which relative tension is more concentrated at the AP-oriented edges. In contrast, MR cells in mutant lenses show isotropic junctional tension on all sides. We conclude that the NMIIA-E1841K mutation results in altered F-actin, NMIIA, N-cadherin, and vinculin distributions, disrupting the anisotropic orientational pattern of mechanical forces within the tissue, leading to disordered cell packing during mouse lens epithelial cell differentiation.

## Abstract (executive summary)

- Nonmuscle myosin IIA (NMIIA) rod domain mutations disrupt rearrangements of eye lens epithelial cells into hexagonally packed meridional row (MR) cells during differentiation, but the mechanism is not known.
- Computational modeling of edge tension and immunolocalization of force sensor proteins (NMIIA, vinculin) demonstrate anisotropic force distribution in MR cells from control lenses, with greater tension and force at anteriorly posteriorly versus equatorially oriented cell edges.
- The NMIIA-E1841K rod domain mutation, which alters bipolar filament assembly, suppresses tension anisotropy, leading to uniform force distribution along all cell edges. NMIIA controls hexagonal cell patterning via regulating tension distribution during lens epithelial cell morphogenesis.

## INTRODUCTION

A spatially stable hexagonal packing configuration of cells is conserved among many epithelial-derived tissues (Edwards and Kiehart, 1996; Classen *et al.*, 2005; Simone and DiNardo, 2010; Gokhin *et al.*, 2012; Ebrahim *et al.*, 2013; Inoue *et al.*, 2016; Finegan *et al.*, 2019; Roshal *et al.*, 2020; von der Emde *et al.*, 2022; Islam *et al.*, 2023; Islam *et al.*, 2024). In some cases, optimized hexagonal packing geometry of cells supports both structural integrity and biological functions of epithelial-derived tissues (Axelrod, 2006; Sugimura and Ishihara, 2013; Curran *et al.*, 2017). For example, proper airflow during flying is made possible by the Drosophila wing's hexagonally packed hairs (Wootton, 1992). In the vertebrate eye, the ordered hexagonal packing of the ocular lens fiber cells is predicted to minimize light scattering (Tardieu, 1988). The lens fiber cells exhibit nearly perfect hexagonal packing, which is established initially in the lens meridional row (MR) cells during lens fiber cell differentiation, with this differentiation process occurring throughout our entire lifetime (Lovicu and Robinson, 2004; Robinson, 2010; Cheng *et al.*, 2013; Shi *et al.*, 2015; Šikić *et al.*, 2017). Due to the lifelong continuous differentiation of hexagonally packed epithelial cells and the accessible location of these hexagonally packed cells on the lens surface, the mouse lens is an excellent vertebrate system to study hexagonal patterning (Cheng *et al.*, 2013; Shi *et al.*, 2015; Bassnett and Šikić, 2017; Šikić *et al.*, 2017).

**FIGURE 1:**
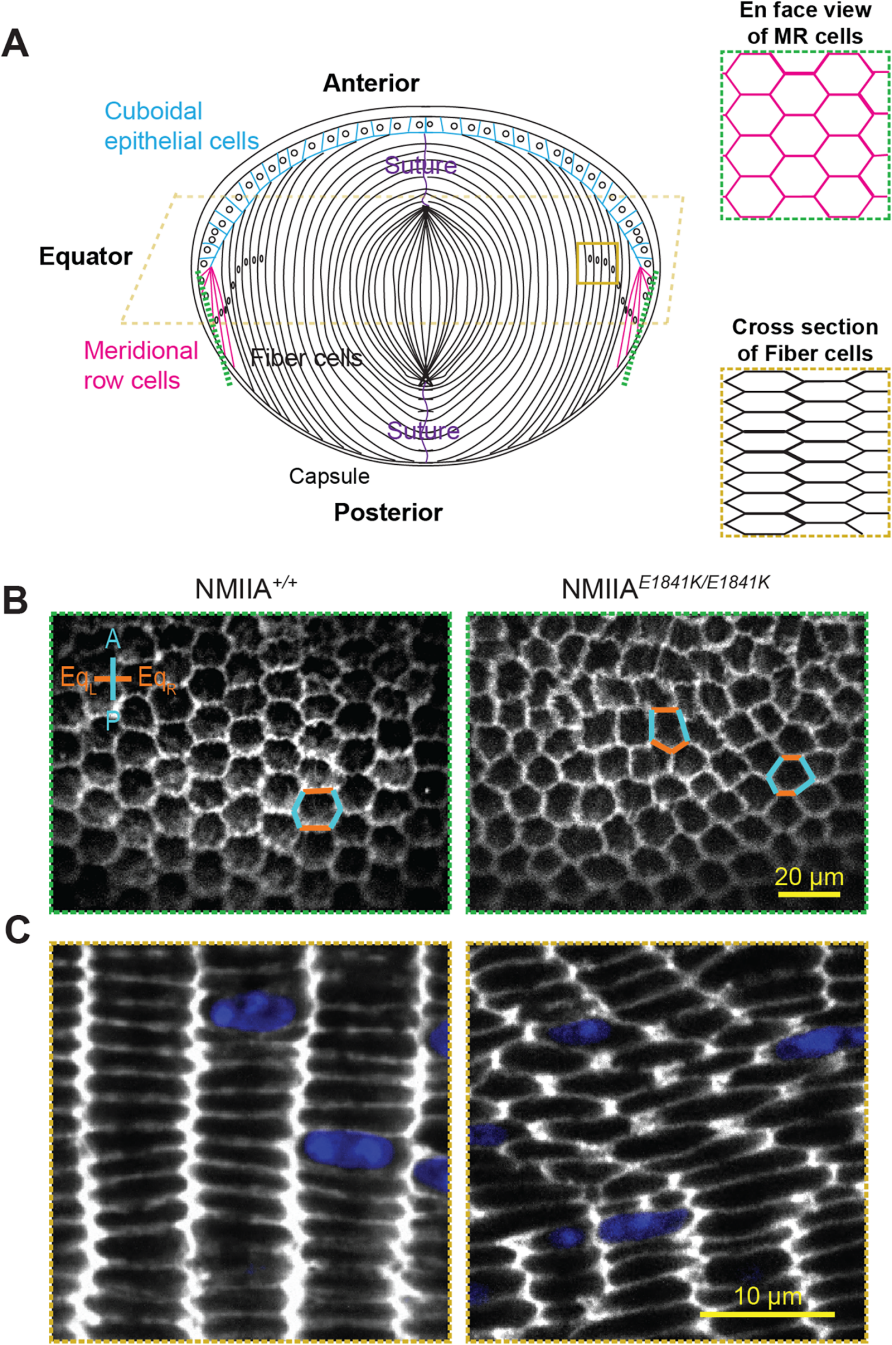
Mouse lens anatomy and lens cellular organization. (A) AP cross-sectional view of mouse lens. The lens is composed of epithelial (blue) and fiber (black) cells. During differentiation, Eq epithelial cells transform into hexagonally packed MR cells (magenta). The green dotted line near the equator in lens diagram and the green dotted box show the *en face* view of MR cells. The yellow dotted box around the lens indicates cross-sectional view and the small yellow box next to MR cells shows newly differentiated (cortical) fiber cells. (B) *En face* view of MR cells labeled for F-actin (gray) close to the surface. MR cells are hexagonally shaped and nearly always have six neighbors in control (NMIIA*^+/+^*, left panel) lenses. NMIIA-E1841K mutation disrupts the hexagon shape and packing of MR cells, as observed in NMIIA*^E1841K/E1841K^* mouse lenses (right panel). Cyan: AP edges, 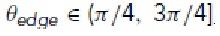; orange: Eq edges, 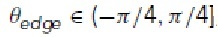; scale bar, 20 µm. (C) Cross-section of cortical fiber cells (small yellow box in A) labeled for F-actin (gray) and nuclei (blue) from NMIIA*^+/+^* (left panel) and NMIIA*^E1841K/E1841K^* lenses (right panel). Fiber cells normally appear as flattened hexagons with six neighbors in the NMIIA*^+/+^* lens (left panel) whereas normal fiber cell packing is disrupted in NMIIA*^E1841K/E1841K^* lens (right panel); scale bar, 10 µm.

The eye lens is a transparent semispherical tissue, surrounded by a basement membrane (capsule), and composed of a monolayer of epithelial cells at the anterior hemisphere and a bulk mass of fiber cells (Figure 1A). The lens epithelial cells near the equator proliferate and differentiate throughout life (Shi *et al.*, 2015; Bassnett and Šikić, 2017; Šikić *et al.*, 2017). At the equator, the randomly packed cuboidal-shaped lens epithelial cells transform into precisely aligned, hexagon-shaped, and hexagonally packed cells to form MR cells, which further differentiate into elongated hexagonally packed secondary fiber cells (Figure 1A, left panel in B, and left panel in C) (Bassnett and Šikić, 2017; Cheng *et al.*, 2017; Šikić *et al.*, 2017; Kalligeraki *et al.*, 2020; Islam *et al.*, 2023). The MR cells form a continuous sheet of hexagonally packed cells around the equatorial (Eq) circumference of the entire lens, in which one set of parallel edges are oriented equatorially around the circumference of the lens (orange edges, Figure 1B) and the other four edges are oriented in the anterior–posterior (AP) direction (cyan edges, Figure 1B) with respect to the optic axis. We previously reported that 98 to 99% of lens MR cells exhibit this remarkable degree of hexagonal patterning (Islam *et al.*, 2023; Islam *et al.*, 2024). How do MR cells establish this nearly perfect hexagonal packing?

Nonmuscle myosin II (NMII) activity is known to regulate cell shape changes and cellular hexagonal patterning during tissue morphogenesis (Edwards and Kiehart, 1996; Bertet *et al.*, 2004; Franke *et al.*, 2005; Major and Irvine, 2006; Montell, 2008; Yamamoto *et al.*, 2009; Monier *et al.*, 2010; Simone and DiNardo, 2010; Ebrahim *et al.*, 2013; Sugimura and Ishihara, 2013; Coravos and Martin, 2016; He *et al.*, 2016; Curran *et al.*, 2017; Agarwal and Zaidel-Bar, 2019; Islam *et al.*, 2023; Islam *et al.*, 2024). NMIIs are motor proteins that self-assemble via their rod domain to form bipolar filaments and pull on oppositely polarized actin filaments (F-actin), generating contractile forces to regulate cell shape, cell–cell contact, cell migration, and cell division (Pollard, 1982; Vicente-Manzanares *et al.*, 2009; Billington *et al.*, 2013; Newell-Litwa *et al.*, 2015). Mutations in the *MYH9* gene coding for NMIIA can cause various conditions in human patients, including thrombocytopenia, kidney disease, hearing loss, liver disease, and cataracts (Pecci *et al.*, 2008; Kunishima and Saito, 2010; Balduini *et al.*, 2011; Pecci *et al.*, 2014; Saposnik *et al.*, 2014; Aoki *et al.*, 2018; Pecci *et al.*, 2018; Tabibzadeh *et al.*, 2019). Three transgenic knockin mouse lines were created, each with a different common human NMIIA mutation: R702C (motor mutation), D1424N (rod mutation), and E1841K (rod mutation) (Zhang *et al.*, 2012; Suzuki *et al.*, 2013). Each mouse line recapitulates most of the phenotypes in human patients, including low platelet counts, kidney defects (proteinuria and glomerulosclerosis), mild hearing loss, and cataracts (Zhang *et al.*, 2012; Suzuki *et al.*, 2013; Cechova *et al.*, 2018; Smith *et al.*, 2019). We previously reported that rod domain mutations (D1424N and E1841K), but not a motor domain mutation (R702C), significantly disrupt lens cellular hexagonal patterning (Islam *et al.*, 2023; Islam *et al.*, 2024). Analysis of 200 MR cells in lenses from NMIIA*^E1841K/E1841K^* mice revealed that ∼33% of the MR cells were irregularly shaped and packed (Figure 1B) (Islam *et al.*, 2023). The altered cell shapes and loss of hexagonal packing persist in the differentiated lens fiber cells of NMIIA*^E1841K/E1841K^* mice, whereas fiber cells in control (NMIIA*^+/+^*) mouse lenses are precisely aligned and hexagonally packed (Figure 1C) (Islam *et al.*, 2023). However, the mechanism through which the NMIIA-E1841K mutation disrupts hexagonal patterning remains unclear.

In this study, we quantified geometric disorder by evaluating over 3000 MR cells from control and mutant lenses. Our analysis reveals a ∼2.5-fold increase in irregularly packed cells in the NMIIA*^E1841K/E1841K^* mice, where many MR cells have five to seven and/or four to eight neighbors rather than the six neighbors of each wild-type hexagonally packed MR cell. We also observe that the normally polarized distribution of NMIIA, N-cadherin, and vinculin at the AP edges in MR cells of control lenses is lost in MR cells of NMIIA*^E1841K/E1841K^* lenses. F-actin is also more concentrated at the Eq edges of MR cells in NMIIA*^E1841K/E1841K^* lenses, compared with a relatively uniform distribution in MR cells of control lenses. Furthermore, we used Bayesian Mechanical Inference of Force to determine how this mutation affects tension distribution (Ishihara and Sugimura, 2012). Our analysis demonstrates that relatively higher tension is associated with the AP-oriented edges compared with the Eq edges in NMIIA*^+/+^* MR cells, while isotropic edge tension is observed for both AP and Eq edges of NMIIA*^E1841K/E1841K^* MR cells. Overall, our findings show that NMIIA is crucial for anisotropic junctional tension that then regulates the hexagonal packing of MR cells.

## RESULTS

### NMIIA-E1841K mutation disrupts mouse lens MR cell packing and shape anisotropy

**FIGURE 2:**
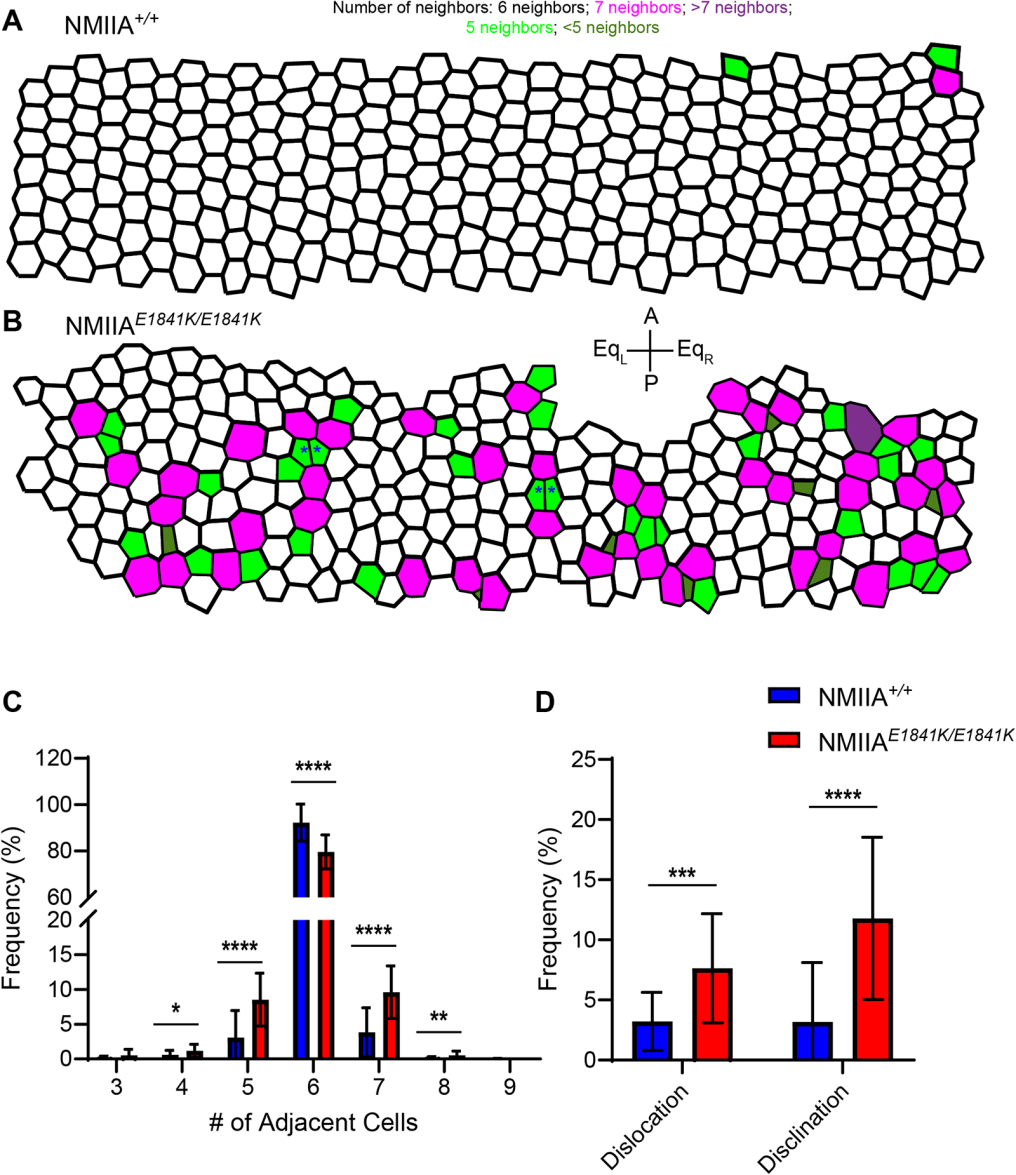
NMIIA-E1841K disrupts the hexagonal packing of MR cells. Representative images of MR cells (boundaries outlined based on F-actin labeling) from (A) NMIIA*^+/+^* and (B) NMIIA*^E1841K/E1841K^* lenses. Black outlined white cells indicate six neighbors. Light magenta indicates seven neighbors, dark magenta indicates more than seven neighbors, light green indicates five neighbors, and dark green indicates less than five neighbors. NMIIA*^E1841K/E1841K^* lens exhibits reduced hexagonal packing. Blue asterisks in pairs of green cells are possible examples of newly formed daughter cells due to delayed or incomplete cytokinesis. (C) Frequency distribution (%) of the number of adjacent cells from NMIIA*^+/+^* and NMIIA*^E1841K/E1841K^* lenses. Plot represents mean ± SD (*N* = 17–26 lens images from at least 10 mice per genotype). (D) Frequency distribution of dislocation and disclination topological defects. Plot represents mean ± SD (*N* = 17–26 lens images from at least 10 mice per genotype). *p < 0.05; **p < 0.01; ***p < 0.001; ****p < 0.0001.

Using F-actin labeling to outline MR cell boundaries at ×20 magnification, we analyzed the numbers of neighbors of 3431 NMIIA*^+/+^* and 5631 NMIIA*^E1841K/E1841K^* MR cells. As shown in Figure 2A, most MR cells in NMIIA*^+/+^* lenses have six neighbors (white cells), although occasionally displaying fewer/more than six neighbors (green/magenta cells). In contrast, NMIIA*^E1841K/E1841K^* lenses show significantly fewer hexagonally packed MR cells, as indicated by an increase in the numbers of green (less than six neighbors) and magenta cells (more than six neighbors) (Figure 2B). The average percentage of MR cells with six neighbors shows that 92.3% ± 8 of NMIIA*^+/+^* cells have six neighbors, while only 79.6% ± 7.4 of NMIIA*^E1841K/E1841K^* MR cells have six neighbors (Figure 2C; Supplemental Table S1). In addition, we observe a significantly increased number of MR cells with four, five, seven, and eight neighbors in NMIIA*^E1841K/E1841K^* lenses compared with NMIIA*^+/+^* lenses (Figure 2C; Supplemental Table S1). Thus, similar to our previous study, we observe a significant decrease in hexagonal packing of MR cells in NMIIA*^E1841K/E1841K^* mouse lenses (Islam *et al.*, 2023).

Cells that do not have six neighbors form two distinct types of topological defects: dislocations and disclinations. Dislocations consist of closely associated pairs of cells with five and seven neighbors, forming clusters of connected defects with an average neighbor number of six and a nonzero Burgers vector. In contrast, disclinations are isolated defects characterized by singular five or seven neighboring cells with an average nearest neighbor number different from six. Although NMIIA*^+/+^* lenses resemble nearly perfect crystals, lacking dislocation and disclination defects, NMIIA*^E1841K/E1841K^* lenses exhibit a pronounced abundance of both types of defects (Figure 2B). In NMIIA*^+/+^* lens MR cells, we observe 3.2 ± 2.4% dislocation and 3.2 ± 4.9% disclination defects. In contrast, the NMIIA*^E1841K/E1841K^* lens MR cells exhibit 7.6 ± 4.5% dislocation and 11.8 ± 6.7% disclination defects, revealing that both types of topological defects are significantly higher in the mutant lenses (Figure 2D). The mutant lenses are more disordered. This spatial organization of topological defects is reminiscent of the melting process in two-dimensional materials (Bowick and Giomi, 2009). NMIIA*^+/+^* behaves in a crystal-like manner, whereas the high density of disclinations in NMIIA*^E1841K/E1841K^* suggests liquid-like states. It should be noted that defect counting tends to be overestimated in small images, especially near boundaries; for example, a 5 to 7 pair may be partially captured, showing only the 5-fold–coordinated site, which could lead to a dislocation being miscounted as a disclination. Likewise, a genuine 5–7–7–5 arrangement, which is not a defect, could be miscounted as a dislocation when only a 5–7 segment is captured at the image boundary. Therefore, we propose using 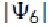 as a more reasonable approach in the current analysis, to be detailed later; however, larger tissue images would likely improve classification accuracy in future studies. In addition to topological defects, we also observed pairs of cells in the NMIIA*^E1841K/E1841K^* lenses that resemble pairs of daughter cells, as indicated by an apparent mirror symmetry in their shapes (Figure 2B, pairs of green cells with asterisks). These cell pairs may derive from a cell with a delayed or incomplete cytokinesis; these cell pairs are not normally observed in NMIIA*^+/+^* lenses.

**FIGURE 3:**
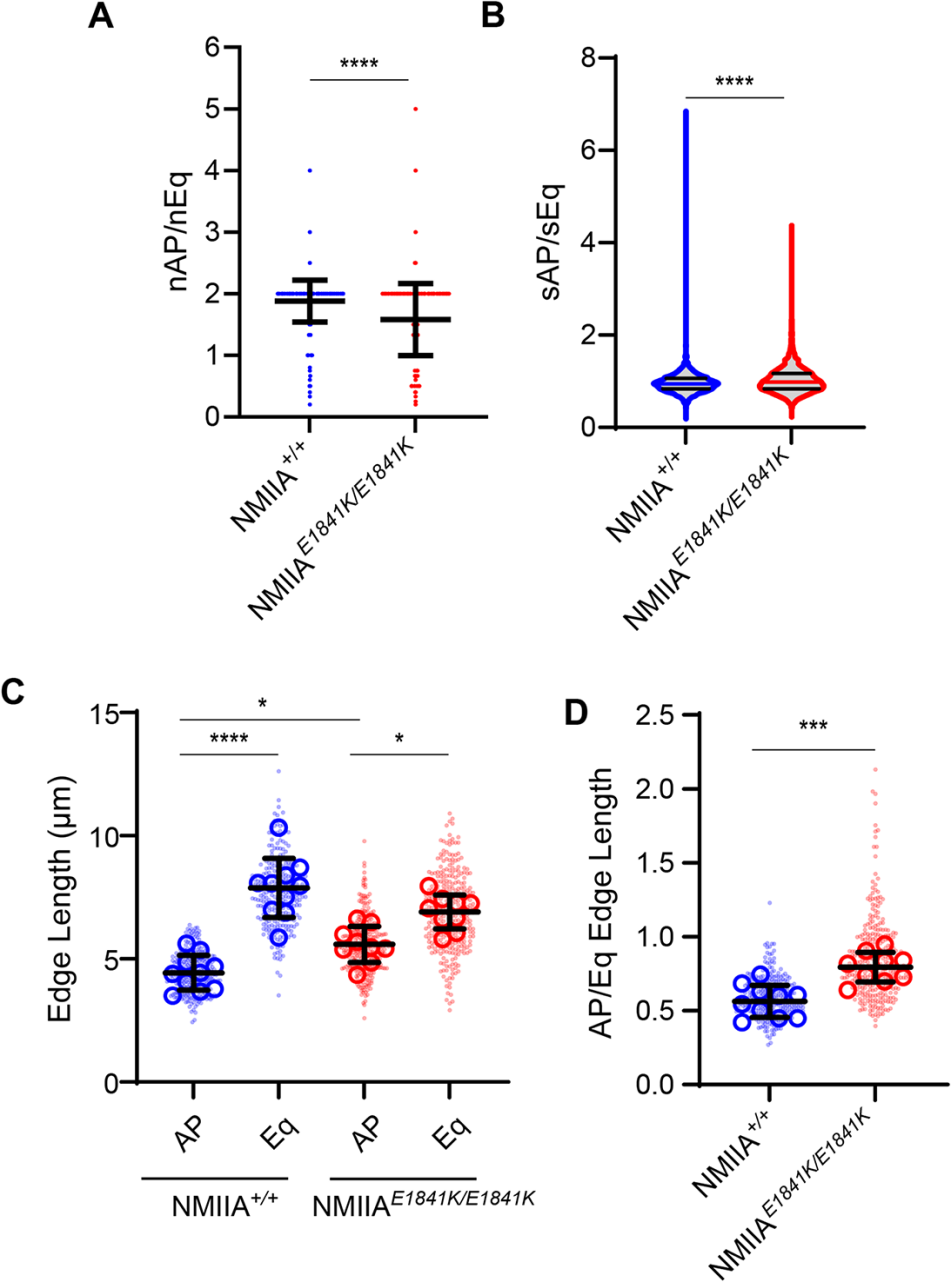
Edges in AP orientation are relatively longer in NMIIA*^E1841K/E1841K^* MR cells. (A) Number (*n*) of AP to Eq edge ratio is lower in the NMIIA*^E1841K/E1841K^* lenses (*N* = 3431-5631 cells). Many NMIIA*^+/+^* and NMIIA*^E1841K/E1841K^* MR cells exhibit nAP/nEq value of two, indicated by significant clustering of blue/red dots that appear as a blue/red line. Plots reflect mean ± SD. (B) Violin plot of MR cell shape anisotropy (S_AP_/S_Eq_) in NMIIA*^+/+^* and NMIIA*^E1841K/E1841K^* lenses, with the median indicated by the straight blue/red lines, whereas the black lines indicate 25th and 75th percentiles (*N* = 3431–5631 cells). (C) The average length of both AP and Eq-oriented edges calculated from images of cells in MR flat mounts. Each bright blue/red open circle represents the average AP or Eq edge length per lens, whereas the dim blue/red dots represent the edge length per cell. (D) The average Ap/Eq edge length is significantly higher in NMIIA*^E1841K/E1841K^* lenses. Each bright blue/red open circle represents the average intensity ratio per lens, whereas dim blue/red dots represent the edge length intensity ratio of a cell. (C and D) Plots reflect mean ± SD (*n* = 9–10 lenses; *N* = 280–340 cells). *p < 0.05; ***p < 0.001; ****p < 0.0001.

As the MR cells are abnormally shaped in the NMIIA*^E1841K/E1841K^* lenses, we also determined the number of AP and Eq edges in both NMIIA*^+/+^* and NMIIA*^E1841K/E1841K^* MR cells. Usually, most NMIIA*^+/+^* MR cells have 4 AP edges and 2 Eq edges, meaning the average ratio of number of AP to Eq (nAP/nEq) edges is 2 for each cell. The average nAP/nEq for NMIIA*^+/+^* MR cells is ∼1.9 ± 0.3 (Figure 3A); while most NMIIA*^+/+^* MR cells have nAP/nEq values of 2 (25th, 50th, and 75th percentiles equal 2), a small percentage of cells exhibit higher or lower nAP/nEq (5th percentile is 1). In comparison, the average nAP/nEq in NMIIA*^E1841K/E1841K^* MR cells is ∼1.6 ± 0.6 (Figure 3A); the 50th and 75th percentile ratio is two while the 25th percentile ratio is one in NMIIA*^E1841K/E1841K^* MR cells, indicating that the average number of AP and Eq edges deviate from four and two edges, respectively, in the NMIIA*^E1841K/E1841K^* MR cells.

Next, we computed the nematic order parameter (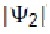) and the hexatic order parameter (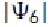) across the tissue to further quantify the order and spatial organization in MR cell packings. These parameters quantify the degree of orientational order in the system, with values ranging from 0 to 1, where higher values indicate stronger alignment and greater orientational coherence (defined in Materials and Methods); nematic and hexatic order values less than 0.5 are considered low, whereas values higher than 0.5 are considered high. The 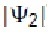 reflects 2-fold symmetry (nematic alignment), characterizing broader anisotropic organization such as elongated cell alignment or filamentary structures. Conversely, 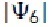 measures the extent of 6-fold (hexagonal) rotational symmetry, providing insight into local cell packing and the influence of hexagonal tiling. Recent studies have demonstrated that epithelial layers exhibit multiscale liquid crystal order, where both hexatic order and nematic order can coexist (Armengol-Collado *et al.*, 2023). We show a schematic in Supplemental Figure S1A to demonstrate the potential interplay of the two types of orientational order. In Supplemental Figure S1B, most of the lens tissues exhibit a low nematic order parameter (
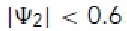
), indicating minimal elongation in cell shapes. The average nematic order for NMIIA*^+/+^* and NMIIA*^E1841K/E1841K^* tissues is 0.3 ± 0.2 and 0.3 ± 0.1, respectively. The average hexatic order value for NMIIA*^+/+^* tissues is 0.8 ± 0.1, whereas the average hexatic order value for NMIIA*^E1841K/E1841K^* tissues is 0.6 ± 0.2, which is significantly lower (*p* < 0.0001). These findings suggest that hexatic order is overall reduced in the NMIIA*^E1841K/E1841K^* MR cells. However, the hexatic order is still relatively dominant in both NMIIA*^+/+^* and NMIIA*^E1841K/E1841K^* mouse lens MR cells, while nematic order is insignificant in either case. The average hexatic values also indicate that NMIIA*^+/+^* lens MR cells are organized similar to a crystal while the average hexatic value in NMIIA*^E1841K/E1841K^* lenses indicate a hexatic phase (Based on our previous observations, crystals typically exhibit |Ψ₆| > 0.65, while hexatic phases generally have |Ψ₆| > 0.35). We have also included examples of the different types of organization we observe in NMIIA*^+/+^* and NMIIA*^E1841K/E1841K^* lens MR cells (Supplemental Figure S1C). Typically, NMIIA*^+/+^* lens MR cells exhibit low 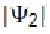 high 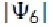 and high 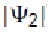 high 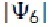, while NMIIA*^E1841K/E1841K^* lens MR cells exhibit low 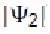 low 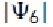 and low 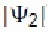 high 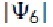. Although a low global nematic order indicates that the MR cells do not exhibit significant collective alignment in a particular direction, they can still become individually elongated. To investigate this, we computed the anisotropy of individual cell shapes (see Materials and Methods). In Figure 3B, we show violin plots of the cell anisotropy ratio, s_AP/s_Eq. A value of one corresponds to unelongated polygonal cells, while higher values indicate elongation in the AP direction, and lower values indicate elongation in the Eq direction. The average s_AP/s_Eq is 0.96 ± 0.22 (25th percentile is 0.83, median is 0.94, and 75th percentile is 1.07) (Figure 3B) in NMIIA*^+/+^* MR cells, overall suggesting elongation predominantly biased toward the Eq direction. In contrast, in NMIIA*^E1841K/E1841K^* MR cells, the average s_AP/ s_Eq is 1.03 ± 0.31 (25th percentile is 0.84, median is 0.98, and 75th percentile is 1.17) (Figure 3B); mean and median values closer to one overall suggests that mutant cells display a more isotropic elongation pattern and lose a preferential elongation bias toward the Eq direction.

As the cell shape anisotropy analysis suggests that NMIIA*^+/+^* MR cells exhibit elongation predominantly biased toward the Eq direction, we calculated the average MR edge length for edges with AP versus Eq orientations in methanol-fixed lens epithelial peels immunolabeled for N-cadherin from NMIIA*^+/+^* and NMIIA*^E1841K/E1841K^* lenses (see Materials and Methods). The average AP edge length of NMIIA*^+/+^* MR cells is 4.4 ± 0.7 µm, while the average Eq edge length is 7.9 ± 1.2 µm (Figure 3C); the average AP to Eq edge length is 0.56 (Figure 3D). The average AP edge length of NMIIA*^E1841K/E1841K^* MR cells is 5.6 ± 0.7 µm and the average Eq edge length is 6.9 ± 0.7 µm (Figure 3C); the average AP to Eq edge length is 0.79 (Figure 3D). These experimental data suggest that the Eq edges are significantly more elongated than AP edges in NMIIA*^+/+^* MR cells, in agreement with the anisotropy calculated for individual cell shapes above. Although Eq edges are still relatively more elongated than AP edges in NMIIA*^E1841K/E1841K^* MR cells, the difference between the AP and Eq edge lengths is smaller in the mutant lenses compared with the control lenses. Our edge length analysis supports the conclusion that although the individual NMIIA*^E1841K/E1841K^* MR cells are more elongated, they have reduced their preferential elongation direction toward the Eq axis compared with control MR cells.

### Cell proliferation and division are not increased in MR cells from NMIIA^E1841K/E1841K^ mice

**FIGURE 4:**
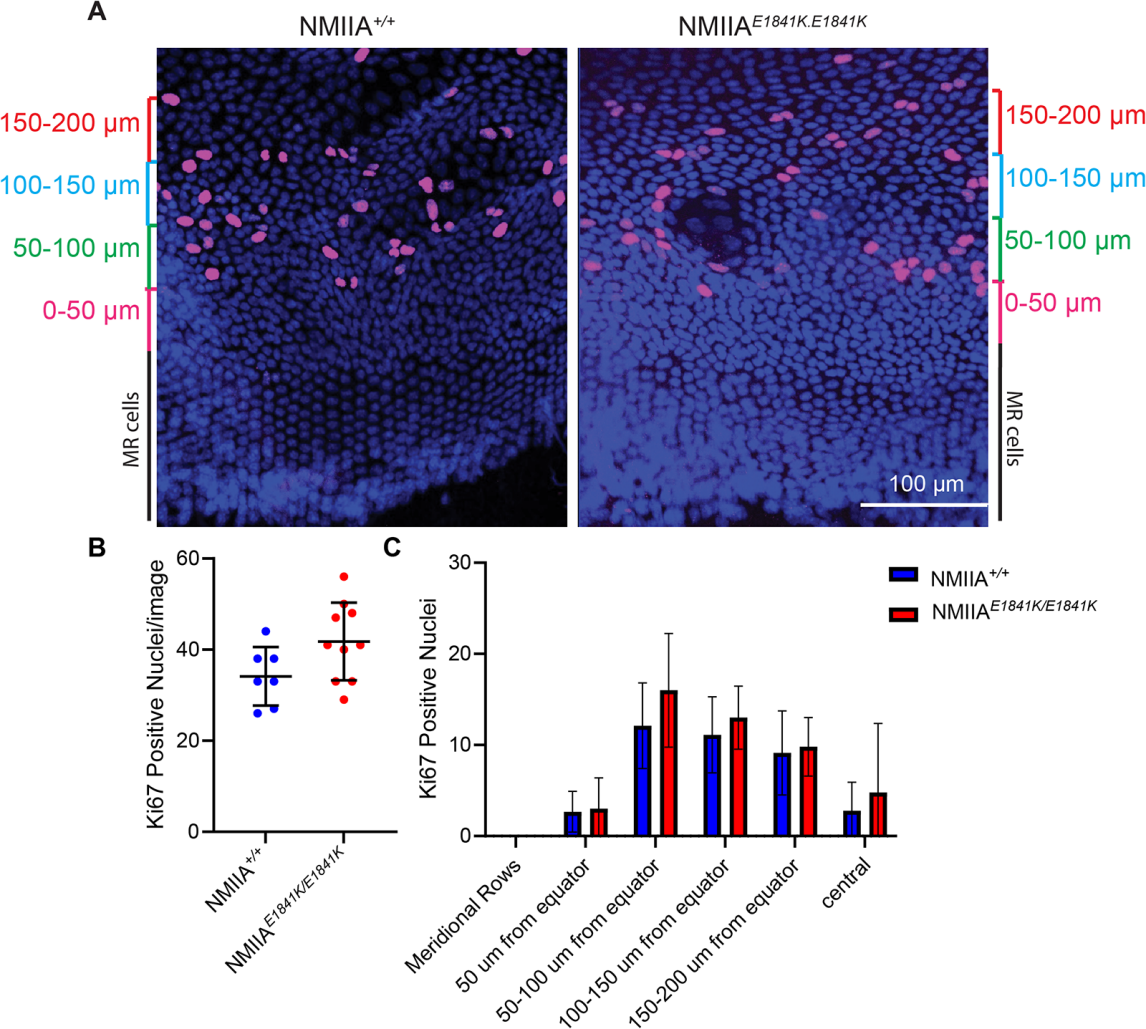
The total number of proliferating cells is unaffected in NMIIA*^E1841K/E1841K^* lenses. (A) Methanol fixed lens epithelial peel labeled for nuclei (blue) and Ki67 (magenta). Ki67-positive nuclei are present in a region 50 to 200 µm anterior to the MR cells; scale bar, 100 µm. (B) The average number of total Ki67-positive cells per image is similar in NMIIA*^+/+^* and NMIIA*^E1841K/E1841K^* lenses. (C) Distribution of Ki67-positive nuclei in different regions. Plots reflect mean (bar) ± SD (*N* = 7–10 lens images from at least three mice per genotype).

Cell division can introduce localized defects in hexagonally packed monolayers of epithelial cells; only a static tissue without proliferation can maintain a perfect hexagonal packing pattern (Farhadifar *et al.*, 2007; Sugimura and Ishihara, 2013). Furthermore, mirror symmetry of some cell pairs in NMIIA*^E1841K/E1841K^* lenses potentially indicates delayed cytokinesis (examples in Figure 2B, blue asterisks in pairs of green cells), potentially suggesting cell division/proliferation defects in the mutant mouse lenses. Normally, very little proliferation occurs in or near MR cells in mature 2-mo-old mouse lenses, in which the infrequent proliferating/dividing cells are located ∼100 µm anterior to the MR cell region (Shi *et al.*, 2015). Because cell proliferation/division rates are significantly greater in younger mouse lenses (Shi *et al.*, 2015), we examined 5-wk-old mouse lenses to determine whether the NMIIA-E1841K mutation may lead to increased epithelial cell proliferation and cell division events near or within the MR region. We labeled lens epithelial cells from 5-wk-old mice for Ki67 (cell proliferation marker) and Hoechst 33342 (nuclei) to determine the number of Ki67-positive nuclei in NMIIA*^+/+^* and NMIIA*^E1841K/E1841K^* lenses (Figure 4A). The total number of Ki67-positive cells is not significantly different between NMIIA*^+/+^* and NMIIA*^E1841K/E1841K^* lenses (Figure 4B), and no Ki67-positive nuclei are observed in the MR regions of either NMIIA*^+/+^* or NMIIA*^E1841K/E1841K^* lenses (Figure 4, A and C). Further analysis of the locations of Ki67-positive cells showed that the region 0 to 50 µm anterior to the MR cells has little to no proliferative activity, while the region 50 to 200 µm anterior to the MR cells has higher proliferative activity in both NMIIA*^+/+^* and NMIIA*^E1841K/E1841K^* lenses, but with no significant differences between them (Figure 4, A and C). We also examined the central epithelium (at least 600 µm anterior to the MR cells), which has little proliferative activity in both control and mutant lenses (Figure 4C), as expected from previous studies (Shi *et al.*, 2015).

Next, we investigated differences in mitotic activity in 4-wk-old control and mutant lenses, by labeling whole lenses for phospho-histone H3 (mitotic marker) and nuclei. Mitotic cells are not observed in MR regions of wild-type lenses and are present in Eq epithelial cell regions immediately anterior to the MR cells, in the same region as the Ki-67–positive proliferating cells (Supplemental Figure S2) (Shi *et al.*, 2015). A comparison of control and mutant lenses indicates that the total number and locations of mitotic cells are similar between the control and mutant lenses (Supplemental Figure S2). Thus, an increased rate of cell proliferation and division near or in the region of the MR cells cannot explain the defects in hexagonal packing observed in the MR cells from mutant lenses.

However, we also noticed that the MR cells appeared relatively smaller, and the cell size appeared more variable in the NMIIA*^E1841K/E1841K^* mice compared with the MR cells in NMIIA*^+/+^* mice (Figures 1B and 2B; Supplemental Figure S3A). Therefore, we measured the average cross-sectional cell area of the MR cells in 2-mo-old control and mutant lenses. The average MR cell area in NMIIA*^+/+^* lenses is 109.0 ± 4.9 µm^2^ whereas the average MR cell area in NMIIA*^E1841K/E1841K^* lenses is 21% smaller at 86.1 ± 12.1 µm^2^ (Supplemental Figure S3B). To determine whether the cells are smaller throughout the entire epithelium, we also determined the average cell area of anterior epithelial cells (Supplemental Figure S3C). The average cross-sectional area of the anterior epithelial cells in NMIIA*^+/+^* lenses is 141.6 ± 14.9 µm^2^ whereas the average area of anterior epithelial cells in NMIIA*^E1841K/E1841K^* lenses is 34% smaller at 93.4 ± 10.2 µm^2^ (Supplemental Figure S3D). In addition, we measured the cell density in both MR and anterior epithelial regions. The average cell density in MR and anterior epithelial cell regions in NMIIA*^+/+^* lenses is 8.9 ± 0.4 and 7.0 ± 0.9 cells/mm^2^, respectively (Supplemental Figure S3, E and F). The cell density of both MR (10.9 ± 0.6 cells/mm^2^) and anterior epithelial (9.9 ± 1.3 cells/mm^2^) cell regions in NMIIA*^E1841K/E1841K^* lenses is significantly higher (Supplemental Figure S3, E and F). As both the lens anterior epithelial and MR cells are smaller and cell density is greater (see Supplemental Figure S3), while the whole-lens sizes are unchanged in the mutant lenses (Islam *et al.*, 2023), yet no increases in proliferation are observed in mutant lenses, (Figure 4; Supplemental Figure S2), it is possible that increased epithelial cell proliferation in the normal location may take place earlier in the development of the mutant lenses (Shi *et al.*, 2015).

### Decreased relative concentration of NMIIA, N-cadherin, vinculin, and F-actin along AP edges of NMIIA^E1841K/E1841K^ MR cells

**FIGURE 5:**
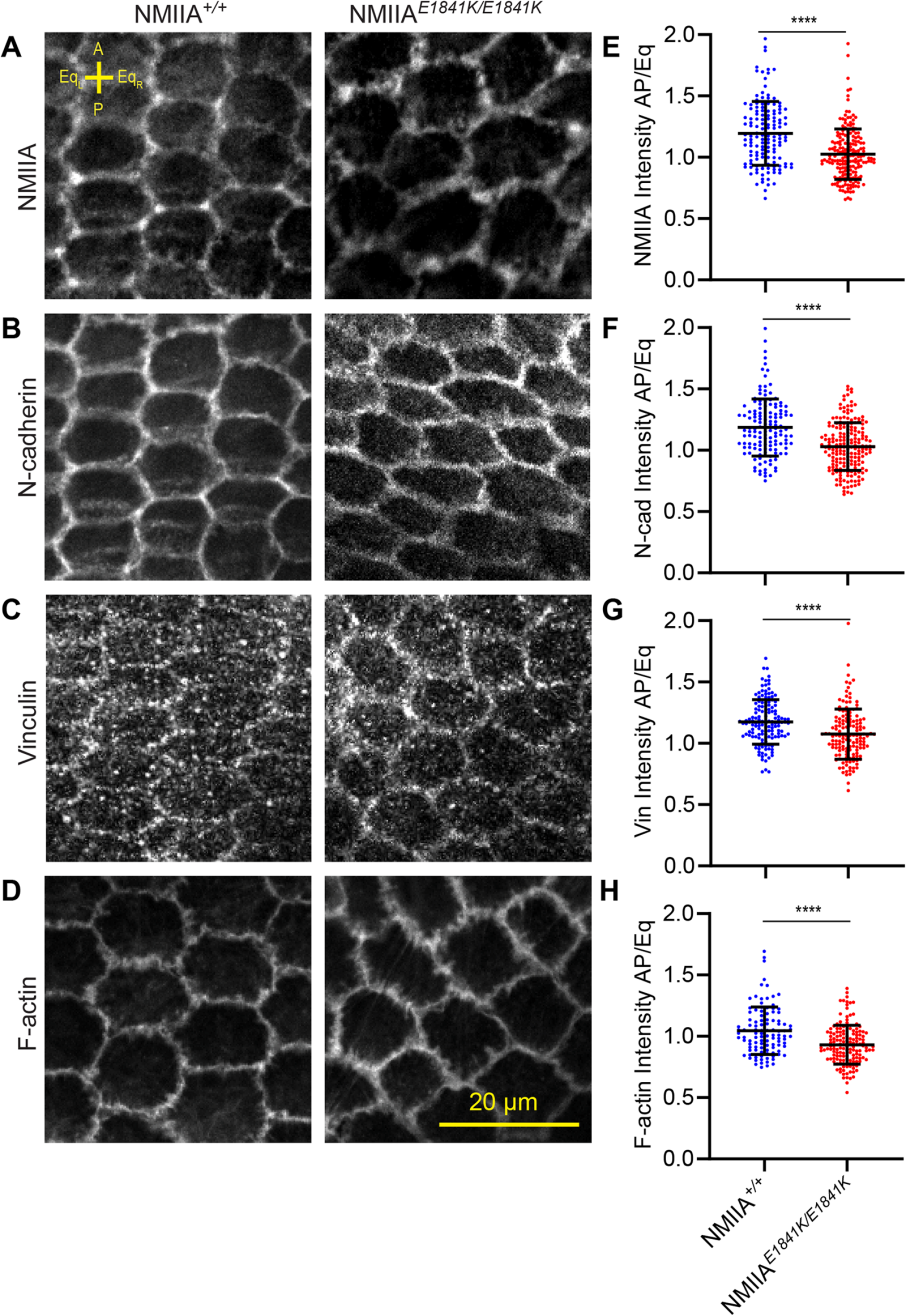
Altered distribution of NMIIA, N-cadherin, vinculin, and F-actin along AP edges of MR cells in NMIIA*^E1841K/E1841K^* compared with NMIIA*^+/+^* MR cells. Methanol-fixed lens epithelial peels labeled for (A) NMIIA, (B) N-cadherin, and (C) Vinculin. (D) Formaldehyde-fixed lens cells labeled with rhodamine-phalloidin for F-actin. (A–D) Representative images show MR cells from NMIIA*^+/+^* and NMIIA*^E1841K/E1841K^* lenses; scale bar, 20 µm. Intensity of (E) NMIIA, (F) N-cadherin, (G) Vinculin, and (H) F-actin at AP versus Eq edges normalized to edge length. The ratio of (E) NMIIA, (F) N-cadherin, (G) Vinculin, and (H) F-actin at AP to Eq edges is significantly lower in NMIIA*^E1841K/E1841K^* lenses compared with NMIIA*^+/+^* lenses. Plots reflect mean ± SD. Each dot represents a cell (*N* of 106–195 cells from at least three mice per genotype). ****p < 0.0001.

The abnormal cell shapes and irregular packing of the lens MR cells in the NMIIA*^E1841K/E1841K^* mouse lenses suggest that cells are not making proper cell-to-cell contacts. NMIIA is a well-known regulator of cell-to-cell contact with either loss or disruption of NMIIA activity causing decreased localization of E-cadherin and vinculin at cell-cell contacts, which is crucial in maintaining junctional stability (Conti *et al.*, 2004; Shewan *et al.*, 2005; le Duc *et al.*, 2010; Ozawa, 2018). Therefore, we investigated the relative localization of contractile and cell-to-cell adhesion proteins at AP versus Eq edges of MR cells from NMIIA*^+/+^* and NMIIA*^E1841K/E1841K^* lenses. We immunolabeled methanol-fixed lens epithelial peels for NMIIA, N-cadherin, and vinculin, followed by confocal fluorescence microscopy (Figure 5A). We specifically looked at N-cadherin instead of E-cadherin, as the MR cells (differentiating fiber cells) upregulate and express N-cadherin at the cell membrane (Leonard *et al.*, 2011; Logan *et al.*, 2017). The intensity of each probe was measured and normalized to edge length along the AP versus Eq edges (Figure 5). An intensity ratio over one would suggest that these proteins are more concentrated at AP edges, a ratio close or equal to 1 that these proteins are equally concentrated at all edges, and a ratio less than one that these proteins are concentrated at Eq edges. The intensity ratios for NMIIA, N-cadherin, and vinculin at AP/Eq edges in NMIIA*^+/+^* MR cells are 1.19 ± 0.26, 1.19 ± 0.23, and 1.18 ± 0.18, respectively (Figure 5, A–C and E–G), suggesting that NMIIA, N-cadherin, and vinculin are somewhat more enriched along the AP edges. In contrast, the intensity ratios for these proteins are significantly lower in the NMIIA*^E1841K/E1841K^* MR cells (*p* < 0.0001). The intensity ratios for NMIIA, N-cadherin, and vinculin at AP/Eq edges in NMIIA*^E1841K/E1841K^* MR cells are 1.03 ± 0.21, 1.03 ± 0.19, and 1.08 ± 0.20, respectively (Figure 5, A–C and E–G). These intensity ratios are close to one in NMIIA*^E1841K/E1841K^* MR cells, suggesting that NMIIA, N-cadherin, and vinculin are equally concentrated along Eq and AP edges.

To detect F-actin along AP and Eq cell edges, we stained whole formaldehyde-fixed lenses with fluorescent phalloidin, and imaged MR cells by confocal fluorescence microscopy (Figure 5D). The average intensity ratio of F-actin at AP versus Eq edges in NMIIA*^+/+^* MR cells is 1.05 ± 0.19 (Figure 5H), indicating that F-actin is relatively equally distributed at both AP and Eq edges in control cells. However, the average intensity ratio of F-actin at AP versus Eq edges significantly decreases in NMIIA*^E1841K/E1841K^* MR cells to 0.93 ± 0.16 (Figure 5H), suggesting less F-actin associates with AP edges while more F-actin associates with Eq edges in mutant MR cells. Overall, our analysis shows an enriched distribution of force sensor proteins at AP versus Eq edges in MR cells in control (NMIIA*^+/+^*) lenses but a relatively more equal distribution of these force sensor proteins at AP and Eq edges in NMIIA*^E1841K/E1841K^* MR cells. This suggests that the NMIIA-E1841K mutation alters junctional tension along AP versus Eq edges in MR cells, disrupting normal cell-to-cell interactions and causing packing defects.

We also measured the mean fluorescent intensity (MFI) of these molecules at NMIIA*^+/+^* and NMIIA*^E1841K/E1841K^* MR cell edges. Although the average MFIs of NMIIA, vinculin, and F-actin are not significantly different, the average N-cadherin MFI is slightly reduced at the NMIIA*^E1841K/E1841K^* MR cell edges (*p* = 0.03) (Supplemental Figure S4). The average MFI values in NMIIA*^+/+^* MR cells for NMIIA, N-cadherin, vinculin, and F-actin are 29 ± 6.4, 21.8 ± 3.8, 13.1 ± 1.1, and 6.1 ± 1.4, respectively (Supplemental Figure S4). The average MFI values in NMIIA*^E1841K/E1841K^* MR cells for NMIIA, N-cadherin, vinculin, and F-actin are 22.4 ± 5.8, 17.0 ± 5.3, 10.6 ± 2.2, and 8.2 ± 3.0, respectively (Supplemental Figure S4). Overall, this may suggest that the MR cell disorder in NMIIA*^E1841K/E1841K^* MR cells is likely not caused by an increase in NMIIA, vinculin, and F-actin levels; rather the altered distribution of these molecules and N-cadherin at MR cell edges is likely a bigger contribution to MR cell disorder in NMIIA*^E1841K/E1841K^* lenses.

### Isotropic tension along the AP and Eq edges in NMIIA^E1841K/E1841K^ MR cells

**FIGURE 6:**
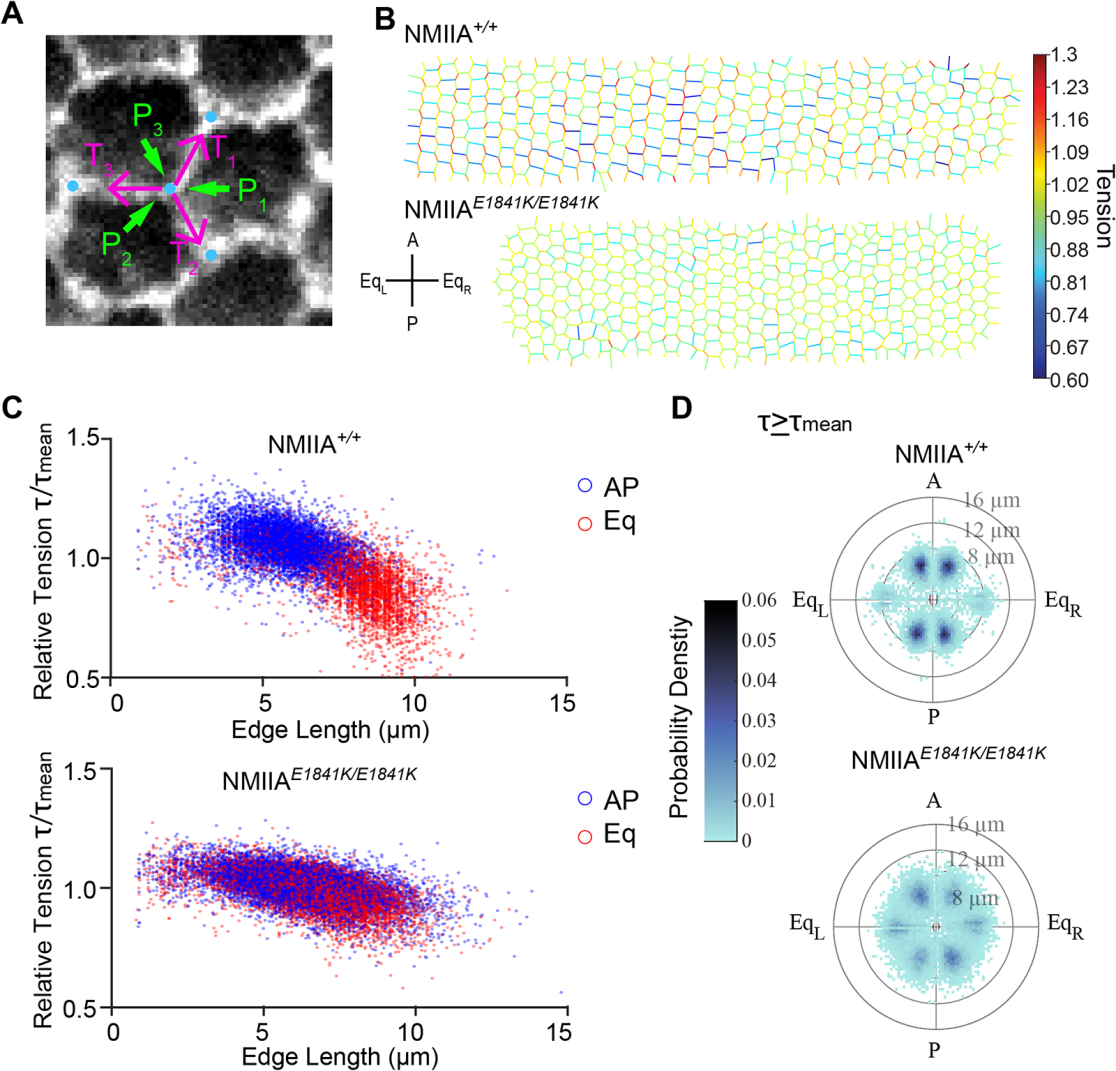
Anisotropic tensions in NMIIA*^+/+^* MR cells contrast with isotropic tensions in NMIIA*^E1841K/E1841K^* cells. (A) Bayesian Method: The tension of each edge and the pressure of each cell are balanced at a vertex. (B) The edge tension for NMIIA*^+/+^* tissue and NMIIA*^E1841K/E1841K^* tissue. Color marks the relative tension value. (C) Negative correlation between relative edge tension and edge length in NMIIA*^+/+^* tissue and NMIIA*^E1841K/E1841K^* tissue, with color, indicating the AP or Eq orientation. (D) Polar distribution of the edge (angle based on direction and length in gray) above mean tension in NMIIA*^+/+^* and NMIIA*^E1841K/E1841K^* lenses.

To test the hypothesis that altered distributions of force sensor proteins such as NMIIA and vinculin along AP versus Eq edges may correspond with altered tensions in MR cells from control versus mutant lenses (Pasapera *et al.*, 2010; Hart *et al.*, 2024), we used Bayesian force inference to solve the inverse problem of estimating cell tensions and pressures (Ishihara and Sugimura, 2012) (see Materials and Methods). This approach assumes that in a mechanically stable confluent tissue, the forces arising from intracellular pressure and junctional tension collectively achieve mechanical force balance at each vertex (Figure 6A). Figure 6B shows the edge tensions obtained using Bayesian force inference in MR cells from both NMIIA*^+/+^* and NMIIA*^E1841K/E1841K^* lenses. These tension maps reveal that, despite cells having similar shapes, the tissues exhibit spatially heterogeneous tension distributions. Notably, both NMIIA*^+/+^* and NMIIA*^E1841K/E1841K^* lens tissues display this heterogeneity, highlighting that even small variations in cell shapes can lead to heterogeneities in tensions.

We further examined the statistical relationships between edge direction, edge length, and edge tension on cellular junctions. A negative correlation between edge tension and edge length was observed, suggesting that shorter edges experience higher tension (Figure 6C). Figure 6C shows that in NMIIA*^+/+^* cells, higher tension values are predominantly associated with edges oriented along the AP direction, while edges in the Eq direction exhibit lower tensions. In contrast, tensions in NMIIA*^E1841K/E1841K^* tissues do not display a strong correlation with edge orientation. To further quantify this tension anisotropy, we plotted the conditional polar distribution of edges with higher-than-mean tension in Figure 6D. In NMIIA*^+/+^* MR cells, the distribution depicts six distinct principal orientations, representing the typical shape of MR cells. High-tension edges are strongly polarized close to the AP axis. However, in NMIIA*^E1841K/E1841K^* lenses, edge orientations are broadly distributed, spanning all directions, and edge lengths vary widely, indicating that tension is not polarized in NMIIA*^E1841K/E1841K^* MR cells. We also plotted the conditional polar distribution of edges with lower-than-mean tensions (Supplemental Figure S5). In NMIIA*^+/+^* MR cells, low-tension edges are strongly polarized along the Eq axis, while the mutant MR cells exhibit a similar pattern as the one with high-than-mean tensions, indicating that tension is not polarized in NMIIA*^E1841K/E1841K^* MR cells (Supplemental Figure S5). Together, these results suggest that NMIIA*^+/+^* MR cells establish a mechanical polarity along the AP direction, whereas NMIIA*^E1841K/E1841K^* MR cells lack such polarity. This directional bias in edge preference is attributed to the interplay between the inherent anisotropy of cell shapes and the tendency for longer edges to possess lower tension.

## DISCUSSION

**FIGURE 7:**
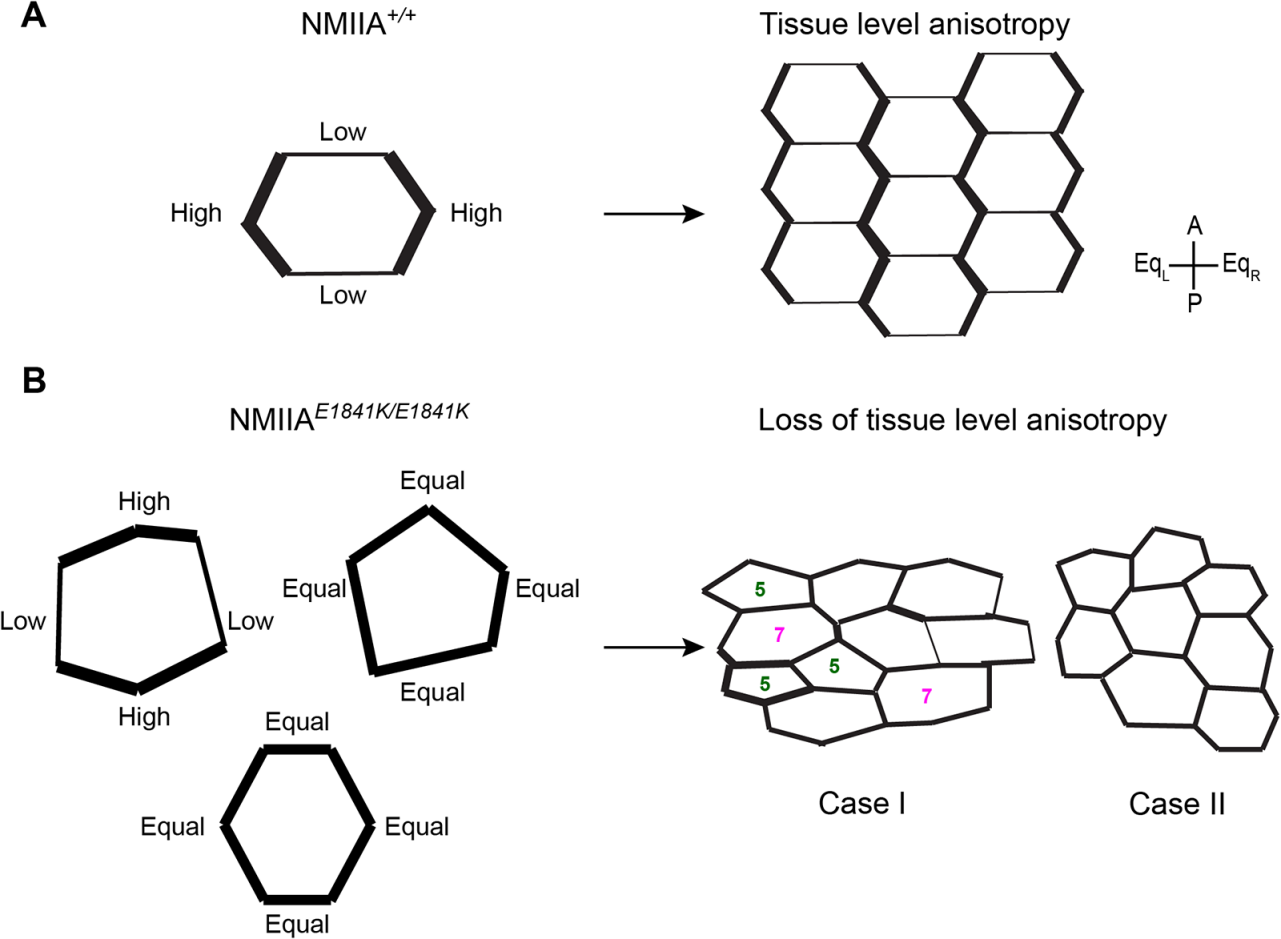
Summary diagram. (A) Distribution of edge tension for NMIIA*^+/+^* MR cell (left) reveals that higher edge tension is localized along the AP axis compared with the Eq axis. The anisotropy of edge tension in the NMIIA*^+/+^* lens MR cells maintains the hexagonal packing at a tissue level (right). (B) Distribution of edge tension for NMIIA*^E1841K/E1841K^* MR cell (left) shows edge tension is not polarized along a particular direction, and/or the edge tension is equally distributed at all orientations. Loss of tension anisotropy disrupts hexagonal patterning, where cells are abnormally shaped and irregularly packed (case I). Even with the loss of polarized tension, we can still observe hexagonal patterning in some regions (case II). The thickness of the edge indicates relative edge tension (thicker edges = relatively higher tension). Nonnumbered cells indicate six neighbors, whereas a magenta number within a cell indicates seven neighbors and a green number within a cell indicates five neighbors. The cartoon is not drawn to scale and does not indicate the relative size of the cells.

The present study aimed to determine the mechanism through which the NMIIA-E1841K mutation disrupts the hexagonal packing of lens MR cells. Here, we report that NMIIA, N-cadherin, and vinculin are relatively more concentrated along the AP-oriented cell edges of lens MR cells in NMIIA*^+/+^* mice, despite relatively equal distributions of F-actin at AP and Eq edges (Figure 5). Computational analysis also reveals increased edge tension at the AP-oriented edges of MR cells in wild-type lenses (Figures 6, C–D, and 7A). In contrast, we observe a relatively reduced association of NMIIA, N-cadherin, vinculin, and F-actin along the AP edges in lens MR cells from NMIIA*^E1841K/E1841K^* mice (Figure 5), as well as reduced tension anisotropy in the MR cells from NMIIA*^E1841K/E1841K^* mice (Figures 6, C–D and 7B), which may explain the irregular cell packing we observed. However, it is worth noting that many mutant MR cells (∼80%) can still exhibit hexagonal packing despite the irregular distribution of tension (Figures 2 and 7B; Supplemental Table S1). Patterning of cells with polarized edge tension along the AP axis may be required to achieve or maintain a perfect hexagonal packing geometry at a tissue level, as in NMIIA*^+/+^* mouse lens MR cells. Because the lens MR cells collectively migrate toward the posterior of the lens to differentiate into fiber cells and elongate apically inwards toward the anterior pole (Lovicu and Robinson, 2004; Robinson, 2010), polarized tension along AP edges may also be necessary to mediate collective cell migration and differentiation of lens MR cells. By contrast, loss of polarized tension in the NMIIA*^E1841K/E1841K^* MR cells appears to introduce occasional packing defects (∼20%; Figure 2C) to meet the isotropic requirement while mostly maintaining some degree of hexagonal packing across the entire tissue.

The role of polarized actomyosin contractile forces in cell shape and packing pattern formation has been studied extensively during *Drosophila* embryonic development and wing formation II (Bertet *et al.*, 2004; Zallen and Wieschaus, 2004; Classen *et al.*, 2005; Blankenship *et al.*, 2006; Rauzi *et al.*, 2008; Ishihara and Sugimura, 2012; Sugimura and Ishihara, 2013). Similar to the lens MR cells, myosin IIs become concentrated along the AP axis compared with the dorsal–ventral axis, generating anisotropic tension and driving cell intercalation during germ band elongation of *Drosophila* embryos (Bertet *et al.*, 2004; Zallen and Wieschaus, 2004; Blankenship *et al.*, 2006; Rauzi *et al.*, 2008; Kasza *et al.*, 2019). Similarly, strong anisotropic stress along the proximal–distal axis compared with the AP axis is required to form hexagonal packing in the *Drosophila* wing (Sugimura and Ishihara, 2013). In this study, we showed that anisotropic edge tension is also a characteristic of hexagonal packing in mouse lens MR cells, in which higher tension is localized along the AP edges compared with the Eq edges with respect to the whole lens (Figures 6 and 7). Immunofluorescence localization of the force sensor protein vinculin (Grashoff *et al.*, 2010; le Duc *et al.*, 2010), which gets recruited to cell junctions under high tension, demonstrates enrichment along the AP edges of lens MR cells, consistent with our computational analysis of edge tension in control lens MR cells. By contrast, computational analysis and immunofluorescence for vinculin shows that the NMIIA-E1841K mutation results in loss of tension anisotropy in lens MR cells. Loss of NMIIA expression and/or activity is known to cause reduced association of E-cadherin, β-catenin, and vinculin with cell junctions in mouse embryos, MDCK cells, and MCF-7 cells, thereby destabilizing cell-to-cell adherens junctions (Conti *et al.*, 2004; Shewan *et al.*, 2005; le Duc *et al.*, 2010; Lecuit, 2010; Ozawa, 2018; Seddiki *et al.*, 2018). In control MR cells, we observe relatively equal distribution of F-actin at AP and Eq edges (Figure 5, D and H), suggesting that polarized F-actin distribution is not required for edge tension anisotropy. In contrast with control MR cells, the NMIIA-E1841K mutation causes reduced F-actin association at AP edges of the mutant MR cells (Figure 5, D and H). However, it is worth noting that we are measuring all F-actin populations at the cell edge, instead of the subset of F-actin that is associated with NMIIA, and attached to the N-cadherin junction. Therefore, the relative distribution of NMIIA-associated F-actin, which also connects to N-cadherin junction, might be relatively more concentrated at the AP edges of control MR cell edges, and our current method does not measure this. Therefore, future work will aim to determine how the NMIIA-E1841K mutation affects the relative distribution of F-actin network associated with NMIIA and N-cadherin at the MR cell edges. Overall, we propose that reduced association of NMIIA observed at AP edges of MR cells in NMIIA*^E1841K/E1841K^* lenses leads to reduced recruitment of N-cadherin, vinculin, and F-actin, contributing to altered tension distribution in the MR cells. Nevertheless, how a NMIIA rod domain mutation may affect contractile activity to generate tension remains unclear.

The NMIIA-E1841K mutation in the rod domain alters normal bipolar filament assembly of NMIIA but does not affect NMIIA ATPase activity in vitro or alter myosin light and heavy chain phosphorylation in megakaryocytes (Kamata *et al.*, 2017; Pal *et al.*, 2020; Casas-Mao *et al.*, 2024). Normally, purified recombinant NMIIA filaments exhibit myosin heads (motor domains) on the opposite ends of the bipolar filaments and a central bare zone where the rod domains interact, lacking myosin heads. In contrast, purified recombinant NMIIA-E1841K filaments lack the central bare zone and display myosin heads along their entire length (Pal *et al.*, 2020). In addition, this mutation causes increased filament thickness and width, possibly indicating the E1841K mutation enhances filament assembly (Pal *et al.*, 2020; Casas-Mao *et al.*, 2024). Paracrystals of the NMIIA-E1841K tail fragments (rod domain) also display aberrant morphology (Franke *et al.*, 2005). However, the NMIIA-E1841K bipolar filament lengths in mouse megakaryocytes and human benign prostate hyperplasia 1 cells do not appear to be significantly different (Pal *et al.*, 2020; Casas-Mao *et al.*, 2024). Interestingly, fluorescence recovery after photobleaching of GFP-tagged NMIIA filaments shows that the NMIIA-E1841K mutation increases the recovery time while reducing the mobile fraction, suggesting that this mutation most likely affects the dynamics of filament formation rather than final filament length in vivo (Casas-Mao *et al.*, 2024). Altered F-actin structures and assembly have been reported in megakaryocytes, testes, and podocytes of mice with E1841K mutations (Cechova *et al.*, 2018; Pal *et al.*, 2020; Sung *et al.*, 2021). Therefore, it is plausible that either altered actomyosin structures or altered dynamics of actomyosin assembly due to the E1841K mutation lead to the redistribution of actomyosin at the MR cell edges, contributing to the remodeling of edge tension and consequent defects in hexagonal packing.

Bayesian force inference (Ishihara and Sugimura, 2012) offers a robust inverse problem framework for quantifying relative forces acting on hundreds or thousands of cells simultaneously at single-cell resolution. By assuming force balance at each vertex, this method allows the determination of relative edge tensions and cell pressures solely from the topology and geometry of cell contacts without requiring assumptions about potential energy forms. Although nonunique solutions may arise from boundary effects of the tissue and non–three-junction configurations, the Bayesian approach incorporating appropriate prior assumptions about tensions, such as gaussian distribution, guides the inference toward physically plausible solutions, even in underdetermined systems. This simplicity makes the method broadly applicable, particularly to static systems (Kong *et al.*, 2019). Even a single image suffices for analysis. Additionally, Bayesian force inference is inherently noninvasive, preserving the integrity of the original experiment and requiring no prior experimental adjustments for force measurement. Note that this method has limitations, such as insufficient temporal resolution for systems exhibiting rapid dynamic changes, which are not the case in the lens. Mechanical perturbation approaches such as laser ablation (Sugimura *et al.*, 2016) are not possible to perform in mammalian organs such as the lens in which the epithelium is surrounded by a thick collagenous capsule (Danysh *et al.*, 2008; Danysh and Duncan, 2009; Cheng *et al.*, 2019). Consequently, Bayesian force inference enables us to infer relative edge tension and provides insight into the role of mechanical force in maintaining a hexagonal packing pattern.

Does cell division and/or proliferation play a role in the hexagonal packing pattern of MR cells? We have previously shown that cell divisions introduce topological defects such as dislocations and dislocations (Tang *et al.*, 2024), as shown in Figure 2B. Furthermore, actomyosin contractile activity has been extensively studied during cell division, particularly for its function in cytokinesis. Both genetic and pharmacological perturbation of myosin IIs, including NMIIA, disrupt cleavage furrow contraction, leading to cytokinesis failure (Mabuchi and Okuno, 1977; De Lozanne and Spudich, 1987; Knecht and Loomis, 1987; Straight *et al.*, 2003; Bao *et al.*, 2005). In our present study, we observe that NMIIA*^+/+^* MR and anterior epithelial cells are ∼1.3 and 1.5 times larger than NMIIA*^E1841K/E1841K^* cells, respectively, while whole-lens size in mutant lenses is similar to control lenses (Figure 4; Supplemental Figures S2 and S3) (Islam *et al.*, 2023). This suggests mutant cells most likely divide faster to generate more smaller-sized cells while keeping the whole-lens size the same as the control lens. Both the rate and location of cell division in eye lens tissue are dependent on age. Cell division is high and occurs throughout the entire epithelium in lenses in Embryonic day 18.5 and 1-wk-old mice (Shi *et al.*, 2013). Cell division slows down significantly with lens maturation and aging and is restricted to a location 100 to 300 µm anterior of the MR cells in mouse lenses older than 2 wk (Shi *et al.*, 2015; Šikić *et al.*, 2017). As we do not see increased cell proliferation and/or more dividing cells near or in MR cell regions of mature NMIIA*^E1841K/E1841K^* mouse lenses (Supplemental Figures S2 and S3), altered rates of cell proliferation and division are unlikely to contribute to irregular packing of MR cells in adult mice. However, we cannot rule out a delay in cytokinesis completion of epithelial cells anterior to the MR cells as we observe pairs of cells with a mirror symmetry in their shapes, which resemble recently formed daughter cells (Figure 2B, blue asterisks in green cells) (Albrecht-Buehler, 1977; Tawk *et al.*, 2007; Ragkousi and Gibson, 2014). Delays in cell division could also lead to local packing defects in MR cells. Future studies will be required to determine whether the NMIIA-E1841K mutation affects cytokinesis rate and/or leads to incomplete or delayed cytokinesis, as our current proliferation/mitosis assay cannot assess cytokinesis defects. Investigating the effect of NMIIA-E1841K mutation on cytokinesis will help determine the relative impact of cytokinesis defects versus altered tension distribution on the loss of hexagonal packing in lens MR cells.

Other than affecting cytokinesis, actomyosin networks also regulate other stages of cell division (Rosenblatt *et al.*, 2004; Matsumura *et al.*, 2011; Sommi *et al.*, 2011; Garrido-Casado *et al.*, 2021). During prophase, myosin IIs assemble at the cortex to regulate cortical tension (Sommi *et al.*, 2011). Actomyosin tensile activities and mechanical cues also drive mitotic spindle positioning, spindle assembly, and the overall orientation of cell division (Rosenblatt *et al.*, 2004; Chanet *et al.*, 2017; Scarpa *et al.*, 2018; Lisica *et al.*, 2022; Blanchard *et al.*, 2024). Some studies have suggested that overall tissue-scale mechanical cues may play a critical role in controlling spindle and cell division orientation (Mao *et al.*, 2011; Wyatt *et al.*, 2015; Nestor-Bergmann *et al.*, 2019). Whether the NMIIA-E1841K mutation affects the overall cell division rate by increasing cytokinesis, and/or cortical tensile activities that regulate the plane of cell division and how these factors may contribute to MR hexagonal patterning remain unclear. In addition, it is not known when hexagonal packing is initially established in the mouse lens during development, and how the high cell division rate in the young lens (mice younger than 2 wk) influences hexagonal patterning of MR cells. Furthermore, the 24-h cell-cycle duration of epithelial cells in mature mouse lenses (Rafferty and Smith, 1976) makes it difficult to determine the effect of NMIIA-E1841K mutation on different stages of cell division using live imaging methods. In the future, it would be advantageous to investigate the effects of the NMIIA-E1841K mutation on cell division and the interplay between cell division and hexagonal pattern formation, using a simple and manipulable model system such as the *Drosophila* wing. This system would also provide an opportunity to experimentally measure the absolute cell edge tension using methods such as laser ablation (Sugimura *et al.*, 2016) and directly determine whether the NMIIA-E1841K mutation disrupts hexagonal cell patterning by affecting cellular tension anisotropy.

The biological significance of lens MR and fiber cell hexagonal patterning is not well understood. Our previous work has shown that loss of hexagonal packing in the *Myh9-*RD mutant mice does not affect whole-lens size, shape, biomechanics, and transparency (Islam *et al.*, 2023; Islam *et al.*, 2024). So, why is the hexagonal patterning of lens cells conserved among different species? The hexagonal cell packing pattern is the most stable configuration on a flat surface as it minimizes surface tension and reduces intracellular space (Thompson, 1917). Thus, it is plausible that packing efficiency is the main reason lens cellular hexagonal packing is conserved, although it may also have another biological function. For instance, hexagonal patterning of lens cells is hypothesized to minimize light scattering (Tardieu, 1988), which is important for clear vision and accurate focusing. Therefore, in future experiments, it will be interesting to measure quantitatively the light scattering properties of lenses from control and NMIIA-E1841K mutant mice; if hexagonal patterning of lens cells plays a role in light scattering properties, we would expect increased light scattering in the NMIIA*^E1841K/E1841K^* mouse lenses. The genetic knockin mice with *Myh9* mutations will provide us with an opportunity to study the relationship between hexagonal patterning and lens optical properties, and may explain aspects of human disease pathology in *MYH9*-RD individuals.

## MATERIALS And METHODS

Request a protocol through *Bio-protocol*

### Mice and materials

All animal procedures were conducted per the ARVO Statement for the Use of Animals in Ophthalmic and Vision Research and approved animal protocols from the Institutional Animal Care and Use Committee guidelines at the University of Delaware. Genetic knockin mice with a *Myh9-*E1841K mutation were obtained from Dr. Robert Adelstein (National Heart, Lung, and Blood Institute, National Institutes of Health, Bethesda, MD) (Zhang *et al.*, 2012). The *Myh9-*E1841K mouse strain was created in an FvBN/129SvEv/C57Bl6 mixed background. The FvBN strain carries an endogenous mutation in the *Bfsp2* gene that causes a spontaneous knockout of CP49 (beaded intermediate filament protein), the loss of which alters lens fiber cell morphology and lens biomechanical properties (Sandilands *et al.*, 2003; Simirskii *et al.*, 2006; Yoon *et al.*, 2008; Gokhin *et al.*, 2012). We backcrossed the mice with C57BL6/J wild-type mice and screened the offspring for the presence of the wild-type CP49 allele. We intercrossed NMIIA*^E1841K/+^* mice to generate NMIIA*^+/+^*, and NMIIA*^E1841K/E1841K^* littermate mice; these mice have been maintained and studied in our laboratory since 2014 (Pal *et al.*, 2020; Islam *et al.*, 2023). Transnetyx performed all genotyping (Cordova, TN).

All materials used in this study are listed in Supplemental Table S2.

### Lens whole-mount staining

Whole-mount staining was performed on freshly dissected lenses from 2-mo-old mice as described previously (Parreno *et al.*, 2018; Islam *et al.*, 2023; Emin *et al.*, 2024; Islam *et al.*, 2024). Whole lenses were fixed by immersing in 4% paraformaldehyde in 1 × PBS at room temperature for 1 h. Next, fixed lenses were washed in 1 × PBS (3  ×  5 min). Lenses were incubated overnight at 4°C in permeabilization/blocking solution (3% BSA, 3% normal goat serum and 0.3% Triton X-100) with rhodamine–phalloidin (1:300) and Hoechst 33342 (1:500) to determine lens cell shape, packing, and area. Next morning, lenses were washed in 1 × PBS (3  ×  5 min/wash). To determine mitotic activity, whole-mount staining was performed on freshly dissected lenses from 4-wk-old mice as described above, with the modification that lenses were incubated overnight in the permeabilization/blocking solution with phosphorylated histone H3 ser10 antibody (Alexa-Fluor 488 conjugate, 1:50) and Hoechst 33342 (1:500).

### Lens whole-mount confocal microscopy to image epithelial and MR cells

The lens MR cells and Eq epithelial cells anterior to the MR cells were imaged using a Zeiss LSM880 confocal microscope. Lenses were immobilized in FluoroDish cell culture dishes within a triangular divot in a thin layer of 4% agarose (in 1 × PBS) that was created using a disposable razor blade (Emin *et al.*, 2024). The whole-mount Z-stacks were acquired using a ×10 objective (NA 0.3, digital zoom of 1.0, and Z-step size of 1.0 µm) to determine mitotic activity.

Z-stack images of lens MR cell images were acquired using a ×20 objective (NA 0.8, digital zoom of 1.0, and Z-step size of 0.5 µm) to determine cell packing. Z-stacks of lens MR cells were acquired using a ×40 oil objective (NA 1.3, digital zoom of 1.0, and Z-step size of 0.25) to determine the F-actin staining intensity at the boundary of MR cells. All images were processed in Zeiss Zen Software (Blue 2.6) for further analysis. For the cell packing analysis with the ×20 objective, 14 to 20 lenses from twelve NMIIA*^+/+^* and NMIIA*^E1841K/E1841K^* mice were used for whole-mount imaging. Single optical sections were examined in *XY* view close to the lens surface immediately underneath the lens capsule (Islam *et al.*, 2023; Islam *et al.*, 2024).

To image the anterior epithelial cells using a Zeiss LSM880 confocal microscope, lenses were placed in FluoroDish cell culture dishes with 1 × PBS, anterior side facing down, as described previously (Parreno *et al.*, 2018; Emin *et al.*, 2024). Z-stacks were acquired using the ×40 oil objective (NA 1.3, digital zoom of 1.0, and Z-step size of 0.25) to evaluate anterior epithelial cell area.

### Lens epithelium flat mount and confocal microscopy

As described previously (Parreno *et al.*, 2022), freshly dissected lenses from 2-mo-old mice were immediately fixed in ice-cold methanol for 45 s. The lens was then transferred to a dissection plate with 1 × PBS with the anterior side of the lens facing down. A cut was made on the posterior side using dissecting scissors and forceps to remove the capsule attached to the epithelium (referred to as lens epithelial peel) from the fiber mass. Next, the peels were then transferred to a 48-well plate. The peels were incubated in a blocking solution containing 3% BSA, 3% normal goat serum, and 0.3% Triton X-100 for 1 h at room temperature. Next, lens peels were labeled with primary antibodies (NMIIA, vinculin, N-cadherin, Ki67) (1:50) diluted in blocking solution overnight at 4°C with gentle rocking. Next morning, the peels were washed in 1 × PBS (3  ×  5 min/wash). The peels were labeled for 2 h with fluorescent-conjugated secondary antibody (Alexa-Fluor 647–conjugated goat anti-rabbit IgG and Alexa-Fluor 555–conjugated goat anti-mouse IgG, 1:200 dilution) and Hoechst 33342 (1:500) at room temperature with gentle rocking. Afterward, the peels were washed in 1 × PBS (3  ×  5 min/wash) and transferred to positively charged adhesive microscope glass slides. ProLong Gold antifade reagent was used to mount coverslips on the slides. Imaging was performed using a Zeiss 880 laser-scanning confocal microscope with ×40 oil objective (NA 1.3, digital zoom of 1.0, and Z-step size of 0.25). Representative data are shown from at least four staining experiments from at least four mice per genotype.

### Image analysis of lens whole mounts and epithelial flat mounts

#### Edge length analysis

Epithelial peel flat mounts were prepared as above, and single optical sections of MR cells from control and mutant lenses (at the *XY* plane with ×40 oil objective) immediately underneath the lens capsule, labeled for N-cadherin, were selected. We determined the edge length using ImageJ to calculate the average edge length (at AP and Eq direction) and AP to Eq edge length ratio. A total of 280 to 340 cells from nine lens images from at least eight mice per genotype were used.

#### Cell proliferation and mitosis analysis

To determine cell proliferative activity, maximum intensity projections were performed on Z-stacks of control and mutant lens MR cells from lens flat mounts labeled for Hoechst 33342 (nuclei) and Ki67 (cell proliferation), and numbers of Ki67-positive nuclei in the MR cell region were counted manually. To determine whether there was increased cell proliferation near MR cells, we manually counted the number of Ki67-positive nuclei in regions 0 to 50, 50 to 100, 100 to 150, and 150 to 200 µm (ROI, 50 × 50 µm for each distance category) above the lens MR cells. We also determined the number of Ki67 cells in the central epithelium located in the center of lens epithelial peels (we defined cells at least ∼600 µm distant to the MR cells as central epithelial cells). We calculated the average number of Ki67-positive nuclei for each of these regions from 7 to 10 lens images from at least three biological replicates per genotype.

Maximum intensity projections were performed on Z-stacks of control and mutant lens Eq epithelial and MR cells from lens whole-mount labeled for Hoechst 33342 (nuclei) and phospho-histone H3 (mitotic marker), imaged with ×10 objective. Phospho-histone H3-positive cells at the lens equator were counted manually.

#### MR Cell area analysis

As described previously (Emin *et al.*, 2024), single optical sections in the *XY* plane from Z-stack confocal images of whole lenses were acquired corresponding to where the middle (lateral) region of the epithelial cells was in focus (×20 objective for MR cells; ×40 oil objective for anterior epithelial cells). Rhodamine-phalloidin labeling of F-actin at the membrane was used to visualize the lateral membranes while Hoechst 33342 was used to visualize nuclei of the lens cells. The mid-lateral region of the MR and epithelial cells, where the nuclei are in focus, was exported as a tiff. A region of interest (ROI) was selected from each image. The total area of the ROI was measured in ImageJ, which was then divided by the total number of nuclei present in the ROI to determine the average cell area from each lens image (Emin *et al.*, 2024). A total of seven to 14 MR images from at least six mice per genotype were analyzed, whereas eight to nine anterior epithelium images from at least five mice per genotype were analyzed.

#### Cell density analysis

As described above (Emin *et al.*, 2024), single optical sections in the XY plane from Z-stack confocal images of whole lenses were acquired corresponding to where the middle (lateral) region of the epithelial cells was in focus (×20 objective for MR cells; ×40 oil objective for anterior epithelial cells). Hoechst 33342 was used to visualize nuclei of the lens cells. The mid-lateral region of the MR and epithelial cells, where the nuclei are in focus, was exported as a tiff. ROI of 9109.6 and 10,846.4 µm^2^ were selected for MR and anterior epithelial cells. Total number of nuclei within the selected ROI was divided by ROI area to measure cell density in cells/µm^2^. We multiplied this value by 1000 to compare cell density as cells/mm^2^.

#### NMIIA, N-cadherin, vinculin, and F-actin intensity measurements of MR cells

Epithelial peel flat mounts were prepared as above, and single optical sections of MR cells from control and mutant lenses (at the *XY* plane with ×40 oil objective) immediately underneath the lens capsule, labeled for NMIIA, N-cadherin, and vinculin were selected. As methanol fixation disrupts F-actin structures and prevents phalloidin binding to F-actin, we used images of formaldehyde-fixed whole lenses labeled with rhodamine–phalloidin as described above. To determine F-actin levels, single optical sections of MR cells from control and mutant lens whole-mount images (at the *XY* plane with ×40 oil objective) close to the lens surface and underneath the lens capsule, were selected.

Using these images from flat mounts and whole mounts, we determined the relative intensity of NMIIA, N-cadherin, vinculin, and F-actin at AP edges with respect to Eq edges. We determined the intensity (integrated density) of these different proteins at each edge and the edge length using ImageJ. We divided the intensity of the proteins at each edge by the respective edge length. Then, the normalized intensity to edge length at AP edges was divided by the normalized intensity to edge length at Eq edges. This ratio indicates the relative concentration of proteins at AP versus Eq edges per 1 µm of edge length (ratio > 1 indicates concentrated at AP edges; ratio = 1 indicates equally concentrated at all edges; ratio <1 indicates concentrated at Eq edges). Using the same images, we also measured the MFI of NMIIA, N-cadherin, vinculin, and F-actin at MR cell edges, using the mean gray value function in ImageJ. We calculated the average MFI of edges per image to calculate average MFI per genotype. A total of 138 to 195 cells from four mice per genotype were used to calculate the relative distributions and MFI of NMIIA, N-cadherin, and vinculin, whereas 106 to 152 cells from at least three to four mice per genotype were used to measure the relative distribution and MFI of F-actin.

### Computational analysis of MR cell images from lens whole mounts

For all computational analysis, images from lens whole mounts were used in which MR cells were labeled for F-actin. We selected single optical sections (*XY* plane with ×20 objective) of MR cells close to the lens surface (immediately beneath the lens capsule) for all of the following analyses.

#### Cell boundary extraction

First, a deep learning-based tool, Cellpose, is used to segment cells, manually adjusted as needed (Stringer *et al.*, 2021; Pachitariu and Stringer, 2022). A series of morphological transformations, such as dilation, erosion, and skeletonization (Haralick and Shapiro, 1992; Lam *et al.*, 1992; Kong and Rosenfeld, 1996; Pratt, 2006) are applied to skeletonize the cell boundaries in confluent tissue, representing cells as polygons. Cell neighbors and the precise identification of shared edges are determined primarily by using a watershed algorithm in conjunction with a region adjacency graph, implemented through the imRAG function in MATLAB (Legland, 2025). Topological defects were counted via a MATLAB code to determine the frequency of dislocations and disclinations. In general, there will be clusters of connected defects, and one must measure the associated average neighbor number of the entire cluster to determine whether they are dislocations or disclinations. A dislocation cluster should also preserve a nonzero Burgers vector, which can be approximated by the displacement vector separating the five and the connected seven. The frequencies of disclinations and dislocations are calculated as the fractions of cells exhibiting each defect type in the tissue.

#### Orientational order: hexatic order and nematic order

The hexatic order parameter (6-fold orientational order) of individual cells (Mickel *et al.*, 2013; Armengol-Collado *et al.*, 2023; Armengol-Collado *et al.*, 2024; Tang *et al.*, 2024) is evaluated by 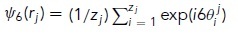, where *z_j_* represents the number of neighboring cells of cell *z* and 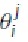 denotes the angle of the joint vector of cell center position (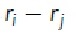) relative to a reference axis. 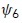 quantifies the uniformity of angle distribution among neighboring cells. A perfect hexagon will have the value of 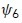 equal to 1. Averaging over all cells in the tissue gives the global hexagonal order parameter 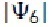, defined as 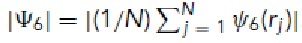. A high 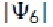 value indicates that the cells not only have neighbors with evenly distributed angles but also exhibit consistent alignment across the entire system.

The nematic order parameter 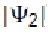
(2-fold orientational order) (Vromans and Giomi, 2016; Tang and Selinger, 2017; Pearce, 2020; Armengol-Collado *et al.*, 2023; Armengol-Collado *et al.*, 2024) is calculated in a similar way but replaced 6 with 2. 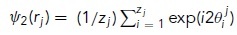 and 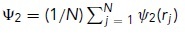.

#### The anisotropy of individual cell shapes

A local cell shape tensor *s_j_* is defined as the sum over all edges of a cell 
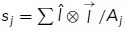
, with the cell edge vector 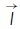 and its unit vector 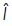. The division by the cell area *A_j_* ensures normalization. The cell anisotropy is quantified by s_AP/s_Eq, where s_AP represents s_yy, capturing alignment along the AP axis, and s_Eq represents s_xx, capturing alignment along the Eq axis.

#### Bayesian force inference

The cell tensions and pressures are estimated by solving an inverse problem within a Bayesian statistical framework (Ishihara and Sugimura, 2012). Force balance equations are constructed at each vertex, as illustrated in Figure 4A. Here, the tension *T* acts along the edge, while the pressure *P* is oriented perpendicular to the line connecting the endpoints of the two adjacent edges. Due to the boundary conditions and four-way junctions, which introduce indefiniteness, the prior distribution of edge tension is assumed to follow a Gaussian distribution with a mean value of one, and observational errors are also modeled as Gaussian-distributed. The mean tension is set to one per image; relative tension values equal to or greater than are considered high tension, whereas relative tension values less than one are considered low tension.

### Statistical analysis

Each experiment was repeated at least three or four times. The sample size (*N*) of each experiment is indicated in the figure legends. Differences between multiple groups of data (edge length) were assessed using one-way ANOVA, whereas differences between two groups of data were assessed using a either unpaired *t* test (for normal distribution) or Mann–Whitney *U* test in GraphPad Prism. All analyses were performed, and graphs were made using GraphPad Prism and Microsoft Excel.

## Supporting information





## Abbreviations used:

NMIIA: nonmuscle myosin IIA
AP: anterior–posterior
Eq: equatorial/equator
MR: meridional row
F-actin: actin filaments
Eq_L_/Eq_R_: equator left/right.

## References

[B1] Agarwal P, Zaidel-Bar R (2019). Principles of actomyosin regulation in vivo. Trends Cell Biol, 29, 150–163. 10.1016/j.tcb.2018.09.006 30385150

[B2] Albrecht-Buehler G (1977). Daughter 3T3 cells. Are they mirror images of each other? J Cell Biol, 72, 595–603. 10.1083/jcb.72.3.595 838769 PMC2111017

[B3] Aoki T, Kunishima S, Yamashita Y, Minamitani K, Ota S (2018). Macrothrombocytopenia with congenital bilateral cataracts: A Phenotype of MYH9 disorder with exon 24 indel mutations. J Pediatr Hematol Oncol, 40, 76–78. 10.1097/MPH.0000000000000998 29200148

[B4] Armengol-Collado J-M, Carenza LN, Eckert J, Krommydas D, Giomi L (2023). Epithelia are multiscale active liquid crystals. Nat Phys, 19, 1773–1779.

[B5] Armengol-Collado JM, Carenza LN, Giomi L (2024). Hydrodynamics and multiscale order in confluent epithelia. Elife, 13. 10.7554/eLife.86400 PMC10963026 38189410

[B6] Axelrod JD (2006). Cell shape in proliferating epithelia: A multifaceted problem. Cell, 126, 643–645. 10.1016/j.cell.2006.07.018 16923381

[B7] Balduini CL, Pecci A, Savoia A (2011). Recent advances in the understanding and management of MYH9-related inherited thrombocytopenias. Br J Haematol, 154, 161–174. 10.1111/j.1365-2141.2011.08716.x 21542825

[B8] Bao J, Jana SS, Adelstein RS (2005). Vertebrate nonmuscle myosin II isoforms rescue small interfering RNA-induced defects in COS-7 cell cytokinesis. J Biol Chem, 280, 19594–19599. 10.1074/jbc.M501573200 15774463

[B9] Bassnett S, Šikić H (2017). The lens growth process. Prog Retin Eye Res, 60, 181–200. 10.1016/j.preteyeres.2017.04.001 28411123 PMC5605917

[B10] Bertet C, Sulak L, Lecuit T (2004). Myosin-dependent junction remodelling controls planar cell intercalation and axis elongation. Nature, 429, 667–671. 10.1038/nature02590 15190355

[B11] Billington N, Wang A, Mao J, Adelstein RS, Sellers JR (2013). Characterization of three full-length human nonmuscle myosin II paralogs. J Biol Chem, 288, 33398–33410. 10.1074/jbc.M113.499848 24072716 PMC3829186

[B12] Blanchard GB, Scarpa E, Muresan L, Sanson B (2024). Mechanical stress combines with planar polarised patterning during metaphase to orient embryonic epithelial cell divisions. Development, 151. 10.1242/dev.202862 PMC11165716 38639390

[B13] Blankenship JT, Backovic ST, Sanny JSP, Weitz O, Zallen JA (2006). Multicellular rosette formation links planar cell polarity to tissue morphogenesis. Dev Cell, 11, 459–470. 10.1016/j.devcel.2006.09.007 17011486

[B14] Bowick MJ, Giomi L (2009). Two-dimensional matter: Order, curvature, and defects. Adv Phys, 58, 449–563.

[B15] Casas-Mao D, Carrington G, Pujol MG, Peckham M (2024). Effects of specific disease mutations in nonmuscle myosin 2A on its structure and function. J Biol Chem, 300, 105514. 10.1016/j.jbc.2023.105514 38042490 PMC10770755

[B16] Cechova S, Dong F, Chan F, Kelley MJ, Ruiz P, Le TH (2018). MYH9 E1841K mutation augments proteinuria and podocyte injury and migration. J Am Soc Nephrol, 29, 155–167. 10.1681/ASN.2015060707 28993503 PMC5748898

[B17] Chanet S, Sharan R, Khan Z, Martin AC (2017). Myosin 2-induced mitotic rounding enables columnar epithelial cells to interpret cortical spindle positioning cues. Curr Biol, 27, 3350–3358.e3. 10.1016/j.cub.2017.09.039 29107549

[B18] Cheng C, Ansari MM, Cooper JA, Gong X (2013). EphA2 and Src regulate equatorial cell morphogenesis during lens development. Development, 140, 4237–4245. 10.1242/dev.100727 24026120 PMC3787762

[B19] Cheng C, Fowler VM, Gong X (2017). EphA2 and ephrin-A5 are not a receptor-ligand pair in the ocular lens. Exp Eye Res, 162, 9–17. 10.1016/j.exer.2017.06.016 28648759 PMC5554726

[B20] Cheng C, Parreno J, Nowak RB, Biswas SK, Wang K, Hoshino M, Yagi N, Moncaster JA, Lo W-K, Moncaster JA, *et al.* (2019). Age-related changes in eye lens biomechanics, morphology, refractive index and transparency. Aging, 11, 12497–12531. 10.18632/aging.102584 31844034 PMC6949082

[B21] Classen A-K, Anderson KI, Marois E, Eaton S (2005). Hexagonal packing of *Drosophila* wing epithelial cells by the planar cell polarity pathway. Dev Cell, 9, 805–817. 10.1016/j.devcel.2005.10.016 16326392

[B22] Conti MA, Even-Ram S, Liu C, Yamada KM, Adelstein RS (2004). Defects in cell adhesion and the visceral endoderm following ablation of nonmuscle myosin heavy chain II-A in mice. J Biol Chem, 279, 41263–41266. 10.1074/jbc.C400352200 15292239

[B23] Coravos JS, Martin AC. (2016). Apical sarcomere-like actomyosin contracts nonmuscle *Drosophila* epithelial cells. Dev Cell, 39, 346–358. 10.1016/j.devcel.2016.09.023 27773487 PMC5102765

[B24] Curran S, Strandkvist C, Bathmann J, de Gennes M, Kabla A, Salbreux G, Baum B (2017). Myosin II controls junction fluctuations to guide epithelial tissue ordering. Dev Cell, 43, 480–492.e6. 10.1016/j.devcel.2017.09.018 29107560 PMC5703647

[B25] Danysh BP, Czymmek KJ, Olurin PT, Sivak JG, Duncan MK (2008). Contributions of mouse genetic background and age on anterior lens capsule thickness. Anat Rec, 291, 1619–1627. 10.1002/ar.20753 PMC2699617 18951502

[B26] Danysh BP, Duncan MK (2009). The lens capsule. Exp Eye Res, 88, 151–164. 10.1016/j.exer.2008.08.002 18773892 PMC2674021

[B27] De Lozanne A, Spudich JA (1987). Disruption of the *Dictyostelium* myosin heavy chain gene by homologous recombination. Science, 236, 1086–1091. 10.1126/science.3576222 3576222

[B28] Ebrahim S, Fujita T, Millis BA, Kozin E, Ma X, Kawamoto S, Baird MA, Davidson M, Yonemura S, Hisa Y, *et al.* (2013). NMII forms a contractile transcellular sarcomeric network to regulate apical cell junctions and tissue geometry. Curr Biol, 23, 731–736. 10.1016/j.cub.2013.03.039 23562268 PMC3658137

[B29] Edwards KA, Kiehart DP (1996). Drosophila nonmuscle myosin II has multiple essential roles in imaginal disc and egg chamber morphogenesis. Development, 122, 1499–1511. 10.1242/dev.122.5.1499 8625837

[B30] Emin G, Islam ST, King RE, Fowler VM, Cheng C, Parreno J (2024). Whole-mount imaging to visualize and quantify peripheral lens structure, cell morphology, and organization. J Vis Exp, 203. 10.3791/66017 PMC11623403 38314859

[B31] Farhadifar R, Röper J-C, Aigouy B, Eaton S, Jülicher F (2007). The influence of cell mechanics, cell–cell interactions, and proliferation on epithelial packing. Curr Biol, 17, 2095–2104. 10.1016/j.cub.2007.11.049 18082406

[B32] Finegan TM, Na D, Cammarota C, Skeeters AV, Nádasi TJ, Dawney NS, Fletcher AG, Oakes PW, Bergstralh DT (2019). Tissue tension and not interphase cell shape determines cell division orientation in the *Drosophila* follicular epithelium. EMBO J, 38. 10.15252/embj.2018100072 PMC6356066 30478193

[B33] Franke JD, Dong F, Rickoll WL, Kelley MJ, Kiehart DP (2005). Rod mutations associated with MYH9-related disorders disrupt nonmuscle myosin-IIA assembly. Blood, 105, 161–169. 10.1182/blood-2004-06-2067 15339844

[B34] Garrido-Casado M, Asensio-Juárez G, Vicente-Manzanares M (2021). Nonmuscle myosin II regulation directs its multiple roles in cell migration and division. Annu Rev Cell Dev Biol, 37, 285–310. 10.1146/annurev-cellbio-042721-105528 34314591

[B35] Gokhin DS, Nowak RB, Kim NE, Arnett EE, Chen AC, Sah RL, John IC, Fowler VM (2012). Tmod1 and CP49 synergize to control the fiber cell geometry, transparency, and mechanical stiffness of the mouse lens. PLoS ONE, 7, e48734. 10.1371/journal.pone.0048734 23144950 PMC3492431

[B36] Grashoff C, Hoffman BD, Brenner MD, Zhou R, Parsons M, Yang MT, McLean MA, Sligar SG, Chen CS, Ha T, *et al.* (2010). Measuring mechanical tension across vinculin reveals regulation of focal adhesion dynamics. Nature, 466, 263–266. 10.1038/nature09198 20613844 PMC2901888

[B37] Haralick RM, Shapiro LG (1992). Computer and Robot Vision, Vol. 1, Addison-Wesley Publishing Company: Reading, MA.

[B38] Hart RG, Kota D, Li F, Zhang M, Ramallo D, Price AJ, Otterpohl KL, Smith SJ, Dunn AR, Huising MO, *et al.* (2024). Myosin II tension sensors visualize force generation within the actin cytoskeleton in living cells. J Cell Sci, 137. 10.1242/jcs.262281 PMC11698044 39369303

[B39] He B, Martin A, Wieschaus E (2016). Flow-dependent myosin recruitment during *Drosophila* cellularization requires zygotic *dunk*activity. Development 10.1242/dev.131334 PMC4958320 27226317

[B40] Inoue Y, Suzuki M, Watanabe T, Yasue N, Tateo I, Adachi T, Ueno N (2016). Mechanical roles of apical constriction, cell elongation, and cell migration during neural tube formation in Xenopus. Biomech Model Mechanobiol, 15, 1733–1746. 10.1007/s10237-016-0794-1 27193152 PMC5106510

[B41] Ishihara S, Sugimura K (2012). Bayesian inference of force dynamics during morphogenesis. J Theor Biol, 313, 201–211. 10.1016/j.jtbi.2012.08.017 22939902

[B42] Islam ST, Cheheltani S, Cheng C, Fowler VM (2024). Disease-related nonmuscle myosin rod domain mutation, but not motor domain mutation, disrupts mouse ocular lens fiber cell alignment and hexagonal packing. Cytoskeleton 10.1002/cm.21853 PMC11416570 38516850

[B43] Islam ST, Cheng C, Parreno J, Fowler VM (2023). Nonmuscle myosin IIA regulates the precise alignment of hexagonal eye lens epithelial cells during fiber cell formation and differentiation. Invest Opthalmol Vis Sci, 64, 20. 10.1167/iovs.64.4.20 PMC10123325 37070941

[B44] Kalligeraki AA, Isted A, Jarrin M, Uwineza A, Pal R, Saunter CD, Girkin JM, Obara B, Quinlan RA. (2020). Three-dimensional data capture and analysis of intact eye lenses evidences emmetropia-associated changes in epithelial cell organization. Sci Rep, 10, 16898. 10.1038/s41598-020-73625-9 33037268 PMC7547080

[B45] Kamata H, Miyazaki K, Komatsu S, Jung H, Craig R, Higashihara M, Suzuki T, Ikebe M (2017). Mutation in the tail region of MYH9 inhibits disassembly of nonmuscle myosin IIA. Kitasato Med, 47, 31–42.

[B46] Kasza KE, Supriyatno S, Zallen JA (2019). Cellular defects resulting from disease-related myosin II mutations in *Drosophila*. Proc Natl Acad Sci USA, 116, 22205–22211. 10.1073/pnas.1909227116 31615886 PMC6825282

[B47] Knecht DA, Loomis WF (1987). Antisense RNA inactivation of myosin heavy chain gene expression in *Dictyostelium discoideum*. Science, 236, 1081–1086. 10.1126/science.3576221 3576221

[B48] Kong T, Rosenfeld A (1996). Topological Algorithms for Digital Image Processing. Elsevier Science Inc: New York.

[B49] Kong W, Loison O, Chavadimane Shivakumar P, Chan EH, Saadaoui M, Collinet C, Lenne P-F, Clément R (2019). Experimental validation of force inference in epithelia from cell to tissue scale. Sci Rep, 9, 14647. 10.1038/s41598-019-50690-3 31601854 PMC6787039

[B51] Kunishima S, Saito H (2010). Advances in the understanding of MYH9 disorders. Curr Opin Hematol, 17, 405–410. 10.1097/MOH.0b013e32833c069c 20601875

[B52] Lam L, Lee S-W, Suen CY (1992). Thinning methodologies-A comprehensive survey. IEEE Trans Pattern Anal Mach Intell, 14, 869–885.

[B53] le Duc Q, Shi Q, Blonk I, Sonnenberg A, Wang N, Leckband D, de Rooij J (2010). Vinculin potentiates E-cadherin mechanosensing and is recruited to actin-anchored sites within adherens junctions in a myosin II-dependent manner. J Cell Biol, 189, 1107–1115. 10.1083/jcb.201001149 20584916 PMC2894457

[B54] Lecuit T (2010). Alpha-catenin mechanosensing for adherens junctions. Nat Cell Biol, 12, 522–524. 10.1038/ncb2066 20453846

[B55] Legland D (2025). Region Adjacency Graph (RAG). MATLAB Central File Exchange. https://www.mathworks.com/matlabcentral/fileexchange/16938-region-adjacency-graph-rag?s_cid=ME_prod_FX.

[B56] Leonard M, Zhang L, Zhai N, Cader A, Chan Y, Nowak RB, Fowler VM, Menko AS. (2011). Modulation of N-cadherin junctions and their role as epicenters of differentiation-specific actin regulation in the developing lens. Dev Biol, 349, 363–377. 10.1016/j.ydbio.2010.10.009 20969840 PMC3018542

[B57] Lisica A, Fouchard J, Kelkar M, Wyatt TPJ, Duque J, Ndiaye AB, Bonfanti A, Baum B, Kabla AJ, Charras GT (2022). Tension at intercellular junctions is necessary for accurate orientation of cell division in the epithelium plane. Proc Natl Acad Sci USA, 119, e2201600119. 10.1073/pnas.2201600119 36454762 PMC7614093

[B58] Logan CM, Rajakaruna S, Bowen C, Radice GL, Robinson ML, Menko AS (2017). N-cadherin regulates signaling mechanisms required for lens fiber cell elongation and lens morphogenesis. Dev Biol, 428, 118–134. 10.1016/j.ydbio.2017.05.022 28552735 PMC5524459

[B59] Lovicu F, Robinson M (2004). Development of the Ocular Lens. Cambridge University Press: Cambridge, UK.

[B60] Mabuchi I, Okuno M (1977). The effect of myosin antibody on the division of starfish blastomeres. J Cell Biol, 74, 251–263. 10.1083/jcb.74.1.251 141455 PMC2109865

[B61] Major RJ, Irvine KD (2006). Localization and requirement for Myosin II at the dorsal–ventral compartment boundary of the *Drosophila* wing. Dev Dyn, 235, 3051–3058. 10.1002/dvdy.20966 17013876

[B62] Mao Y, Tournier AL, Bates PA, Gale JE, Tapon N, Thompson BJ (2011). Planar polarization of the atypical myosin Dachs orients cell divisions in *Drosophila*. Genes Dev, 25, 131–136. 10.1101/gad.610511 21245166 PMC3022259

[B63] Matsumura F, Yamakita Y, Yamashiro S (2011). Myosin light chain kinases and phosphatase in mitosis and cytokinesis. Arch Biochem Biophys, 510, 76–82. 10.1016/j.abb.2011.03.002 21396909 PMC3114266

[B64] Mickel W, Kapfer SC, Schröder-Turk GE, Mecke K (2013). Shortcomings of the bond orientational order parameters for the analysis of disordered particulate matter. J Chem Phys, 138, 044501. 10.1063/1.4774084 23387599

[B65] Monier B, Pélissier-Monier A, Brand AH, Sanson B (2010). An actomyosin-based barrier inhibits cell mixing at compartmental boundaries in Drosophila embryos. Nat Cell Biol, 12, 60–65. 10.1038/ncb2005 19966783 PMC4016768

[B66] Montell DJ (2008). Morphogenetic cell movements: Diversity from modular mechanical properties. Science, 322, 1502–1505. 10.1126/science.1164073 19056976

[B67] Nestor-Bergmann A, Stooke-Vaughan GA, Goddard GK, Starborg T, Jensen OE, Woolner S (2019). Decoupling the roles of cell shape and mechanical stress in orienting and cueing epithelial mitosis. Cell Rep, 26, 2088–2100.e4. 10.1016/j.celrep.2019.01.102 30784591 PMC6381790

[B68] Newell-Litwa KA, Horwitz R, Lamers ML (2015). Nonmuscle myosin II in disease: Mechanisms and therapeutic opportunities. Dis Model Mech, 8, 1495–1515. 10.1242/dmm.022103 26542704 PMC4728321

[B69] Ozawa M (2018). Nonmuscle myosin IIA is involved in recruitment of apical junction components through activation of α-catenin. Biol Open, 7. 10.1242/bio.031369 PMC5992523 29654115

[B70] Pachitariu M, Stringer C (2022). Cellpose 2.0: How to train your own model. Nat Methods, 19, 1634–1641. 10.1038/s41592-022-01663-4 36344832 PMC9718665

[B71] Pal K, Nowak R, Billington N, Liu R, Ghosh A, Sellers JR, Fowler VM (2020). Megakaryocyte migration defects due to nonmuscle myosin IIA mutations underlie thrombocytopenia in MYH9-related disease. Blood, 135, 1887–1898. 10.1182/blood.2019003064 32315395 PMC7243143

[B72] Parreno J, Cheng C, Nowak RB, Fowler VM (2018). The effects of mechanical strain on mouse eye lens capsule and cellular microstructure. Mol Biol Cell, 29, 1963–1974. 10.1091/mbc.E18-01-0035 30088796 PMC6232967

[B73] Parreno J, Emin G, Vu MP, Clark JT, Aryal S, Patel SD, Cheng C (2022). Methodologies to unlock the molecular expression and cellular structure of ocular lens epithelial cells. Front Cell Dev Biol, 10, 983178. 10.3389/fcell.2022.983178 36176273 PMC9514789

[B74] Pasapera AM, Schneider IC, Rericha E, Schlaepfer DD, Waterman CM (2010). Myosin II activity regulates vinculin recruitment to focal adhesions through FAK-mediated paxillin phosphorylation. J Cell Biol, 188, 877–890. 10.1083/jcb.200906012 20308429 PMC2845065

[B75] Pearce DJG (2020). Defect order in active nematics on a curved surface. New J Phys, 22, 063051.

[B76] Pecci A, Klersy C, Gresele P, Lee KJD, De Rocco D, Bozzi V, Russo G, Heller PG, Loffredo G, Ballmaier M, Savoia A (2014). *MYH9*-related disease: A novel prognostic model to predict the clinical evolution of the disease based on genotype-phenotype correlations. Hum Mutat, 35, 236–247. 10.1002/humu.22476 24186861 PMC6233870

[B77] Pecci A, Ma X, Savoia A, Adelstein RS (2018). MYH9: Structure, functions and role of nonmuscle myosin IIA in human disease. Gene, 664, 152–167. 10.1016/j.gene.2018.04.048 29679756 PMC5970098

[B78] Pecci A, Panza E, Pujol-Moix N, Klersy C, Di Bari F, Bozzi V, Gresele P, Lethagen S, Fabris F, Dufour C, *et al.* (2008). Position of nonmuscle myosin heavy chain IIA (NMMHC-IIA) mutations predicts the natural history ofMYH9-related disease. Hum Mutat, 29, 409–417. 10.1002/humu.20661 18059020

[B79] Pollard TD (1982). Structure and polymerization of Acanthamoeba myosin-II filaments. J Cell Biol, 95, 816–825. 10.1083/jcb.95.3.816 7153247 PMC2112911

[B80] Pratt WK (2006). Digital image processing: PIKS Scientific inside, Vol. 4. Wiley Online Library: Los Altos, California.

[B81] Rafferty NS, Smith R (1976). Analysis of cell populations of normal and injured mouse lens epithelium. I. Cell cycle. Anat Rec, 186, 105–113.

[B82] Ragkousi K, Gibson MC (2014). Cell division and the maintenance of epithelial order. J Cell Biol, 207, 181–188. 10.1083/jcb.201408044 25349258 PMC4210436

[B83] Rauzi M, Verant P, Lecuit T, Lenne PF (2008). Nature and anisotropy of cortical forces orienting *Drosophila* tissue morphogenesis. Nat Cell Biol, 10, 1401–1410. 10.1038/ncb1798 18978783

[B84] Robinson ML (2010). Lens fiber cell differentiation. Encyclopedia of the Eye. Elsevier, 543–550. 10.1016/B978-0-12-374203-2.00025-7

[B85] Rosenblatt J, Cramer LP, Baum B, McGee KM (2004). Myosin II-dependent cortical movement is required for centrosome separation and positioning during mitotic spindle assembly. Cell, 117, 361–372. 10.1016/s0092-8674(04)00341-1 15109496

[B86] Roshal DS, Azzag K, Le Goff E, Rochal SB, Baghdiguian S (2020). Crystal-like order and defects in metazoan epithelia with spherical geometry. Sci Rep, 10, 7652–7652. 10.1038/s41598-020-64598-w 32376904 PMC7203251

[B87] Sandilands A, Prescott AR, Wegener A, Zoltoski RK, Hutcheson AM, Masaki S, Kuszak JR, Quinlan RA (2003). Knockout of the intermediate filament protein CP49 destabilises the lens fibre cell cytoskeleton and decreases lens optical quality, but does not induce cataract. Exp Eye Res, 76, 385–391. 10.1016/S0014-4835(02)00330-5 12573667

[B88] Saposnik B, Binard S, Fenneteau O, Nurden A, Nurden P, Hurtaud-Roux MF, Schlegel N (2014). Mutation spectrum and genotype-phenotype correlations in a large French cohort of MYH9-related disorders. Mol Genet Genomic Med, 2, 297–312. 10.1002/mgg3.68 25077172 PMC4113270

[B89] Scarpa E, Finet C, Blanchard GB, Sanson B (2018). Actomyosin-driven tension at compartmental boundaries orients cell division independently of cell geometry in vivo. Dev Cell, 47, 727–740.e6. 10.1016/j.devcel.2018.10.029 30503752 PMC6302072

[B90] Seddiki R, Narayana GHNS, Strale PO, Balcioglu HE, Peyret G, Yao M, Le AP, Lim CT, Yan J, Ladoux B, Mège RM (2018). Force-dependent binding of vinculin to α-catenin regulates cell–cell contact stability and collective cell behavior. Mol Biol Cell, 29, 380–388. 10.1091/mbc.E17-04-0231 29282282 PMC6014167

[B91] Shewan AM, Maddugoda M, Kraemer A, Stehbens SJ, Verma S, Kovacs EM, Yap AS (2005). Myosin 2 is a key Rho kinase target necessary for the local concentration of E-cadherin at cell–cell contacts. Mol Biol Cell, 16, 4531–4542. 10.1091/mbc.e05-04-0330 16030252 PMC1237062

[B92] Shi Y, De Maria A, Lubura S, iki H, Bassnett S (2015). The Penny Pusher: A cellular model of lens growth. Invest Ophthalmol Vis Sci, 56, 799–809. 10.1167/iovs.14-16028 PMC4313793 25515574

[B93] Shi Y, Tu Y, De Maria A, Mecham RP, Bassnett S (2013). Development, composition, and structural arrangements of the Ciliary Zonule of the mouse. Invest Opthalmol Vis Sci, 54, 2504–2504. 10.1167/iovs.13-11619 PMC3621578 23493297

[B119] Šikić H, Shi Y, Lubura S, Bassnett S (2017). A full lifespan model of vertebrate lens growth. R Soc Open Sci, 4, 160695. 10.1098/rsos.160695 28280571 PMC5319337

[B94] Simirskii VN, Lee RS, Wawrousek EF, Duncan MK (2006). Inbred FVB/N mice are mutant at the *cp49/Bfsp2* locus and lack beaded filament proteins in the lens. Invest Opthalmol Vis Sci, 47, 4931–4931. 10.1167/iovs.06-0423 17065509

[B95] Simone RP, DiNardo S (2010). Actomyosin contractility and discs large contribute to junctional conversion in guiding cell alignment within the *Drosophila* embryonic epithelium. Development, 137, 1385–1394. 10.1242/dev.048520 20332153 PMC2847470

[B96] Smith AS, Pal K, Nowak RB, Demenko A, Zaninetti C, Da Costa L, Favier R, Pecci A, Fowler VM (2019). *MYH9*-related disease mutations cause abnormal red blood cell morphology through increased myosin-actin binding at the membrane. Am J Hematol, 94, 667–677. 10.1002/ajh.25472 30916803 PMC6510596

[B97] Sommi P, Cheerambathur D, Brust-Mascher I, Mogilner A (2011). Actomyosin-dependent cortical dynamics contributes to the prophase force-balance in the early *Drosophila* embryo. PLoS ONE, 6, e18366. 10.1371/journal.pone.0018366 21483831 PMC3069073

[B98] Straight AF, Cheung A, Limouze J, Chen I, Westwood NJ, Sellers JR, Mitchison TJ (2003). Dissecting temporal and spatial control of cytokinesis with a myosin II inhibitor. Science, 299, 1743–1747. 10.1126/science.1081412 12637748

[B99] Stringer C, Wang T, Michaelos M, Pachitariu M (2021). Cellpose: A generalist algorithm for cellular segmentation. Nat Methods, 18, 100–106. 10.1038/s41592-020-01018-x 33318659

[B100] Sugimura K, Ishihara S (2013). The mechanical anisotropy in a tissue promotes ordering in hexagonal cell packing. Development, 140, 4091–4101. 10.1242/dev.094060 24046322

[B101] Sugimura K, Lenne PF, Graner F (2016). Measuring forces and stresses in situ in living tissues. Development, 143, 186–196. 10.1242/dev.119776 26786209

[B102] Sung DC, Ahmad M, Lerma Cervantes CB, Zhang Y, Adelstein RS, Ma X (2021). Mutations in nonmuscle myosin 2A disrupt the actomyosin cytoskeleton in Sertoli cells and cause male infertility. Dev Biol, 470, 49–61. 10.1016/j.ydbio.2020.11.003 33188738 PMC7855486

[B103] Suzuki N, Kunishima S, Ikejiri M, Maruyama S, Sone M, Takagi A, Ikawa M, Okabe M, Kojima T, Saito H, *et al.* (2013). Establishment of mouse model of MYH9 disorders: Heterozygous R702C mutation provokes macrothrombocytopenia with leukocyte inclusion bodies, renal glomerulosclerosis and hearing disability. PLoS ONE, 8, e71187. 10.1371/journal.pone.0071187 23976996 PMC3748045

[B104] Tabibzadeh N, Fleury D, Labatut D, Bridoux F, Lionet A, Jourde-Chiche N, Vrtovsnik F, Schlegel N, Vanhille P (2019). MYH9-related disorders display heterogeneous kidney involvement and outcome. Clin Kidney J, 12, 494–502. 10.1093/ckj/sfy117 31384440 PMC6671426

[B105] Tang X, Selinger JV (2017). Orientation of topological defects in 2D nematic liquid crystals. Soft Matter, 13, 5481–5490. 10.1039/c7sm01195d 28785753

[B106] Tang Y, Chen S, Bowick MJ, Bi D (2024). Cell division and motility enable hexatic order in biological tissues. Phys Rev Lett, 132, 218402. 10.1103/PhysRevLett.132.218402 38856284 PMC11267118

[B107] Tardieu A (1988). Eye lens proteins and transparency: From light transmission theory to solution X-ray structural analysis. Annu Rev Biophys Chem, 17, 47–70. 10.1146/annurev.bb.17.060188.000403 3293596

[B108] Tawk M, Araya C, Lyons DA, Reugels AM, Girdler GC, Bayley PR, Hyde DR, Tada M, Clarke JD (2007). A mirror-symmetric cell division that orchestrates neuroepithelial morphogenesis. Nature, 446, 797–800. 10.1038/nature05722 17392791

[B109] Thompson SDAW (1917). On Growth and Form. Cambridge.

[B110] Vicente-Manzanares M, Ma X, Adelstein RS, Horwitz AR (2009). Nonmuscle myosin II takes centre stage in cell adhesion and migration. Nat Rev Mol Cell Biol, 10, 778–790. 10.1038/nrm2786 19851336 PMC2834236

[B111] von der Emde L, Vaisband M, Hasenauer J, Bourauel L, Bermond K, Saßmannshausen M, Heintzmann R, Holz FG, Curcio CA, Sloan KR, Ach T (2022). Histologic cell shape descriptors for the retinal pigment epithelium in age-related macular degeneration: A comparison to unaffected eyes. Transl Vis Sci Technol, 11, 19–19. – 10.1167/tvst.11.8.19 PMC9419462 35984669

[B112] Vromans AJ, Giomi L (2016). Orientational properties of nematic disclinations. Soft Matter, 12, 6490–6495. 10.1039/c6sm01146b 27418339

[B113] Wootton RJ (1992). Functional morphology of insect wings. Ann Rev Entomol, 37, 113–140.

[B114] Wyatt TP, Harris AR, Lam M, Cheng Q, Bellis J, Dimitracopoulos A, Kabla AJ, Charras GT, Baum B (2015). Emergence of homeostatic epithelial packing and stress dissipation through divisions oriented along the long cell axis. Proc Natl Acad Sci USA, 112, 5726–5731. 10.1073/pnas.1420585112 25908119 PMC4426437

[B115] Yamamoto N, Okano T, Ma X, Adelstein RS, Kelley MW (2009). Myosin II regulates extension, growth and patterning in the mammalian cochlear duct. Development, 136, 1977–1986. 10.1242/dev.030718 19439495 PMC2685721

[B116] Yoon K-H, Blankenship T, Shibata B, FitzGerald PG. (2008). Resisting the effects of aging: A function for the fiber cell beaded filament. Invest Opthal Vis Sci, 49, 1030. 10.1167/iovs.07-1149 PMC6746185 18326727

[B117] Zallen JA, Wieschaus E (2004). Patterned gene expression directs bipolar planar polarity in *Drosophila*. Dev Cell, 6, 343–355. 10.1016/S1534-5807(04)00060-7 15030758

[B118] Zhang Y, Conti MA, Malide D, Dong F, Wang A, Shmist YA, Liu C, Zerfas P, Daniels MP, Chan C-C, *et al.* (2012). Mouse models of MYH9-related disease: Mutations in nonmuscle myosin II-A. Blood, 119, 238–250. 10.1182/blood-2011-06-358853 21908426 PMC3251230

