# Supplementary material for: Nonmuscle myosin IIA (NMIIA) regulates anisotropic cell tension to maintain the hexagonal packing of mouse lens meridional row cells
Source: Mol Biol Cell. 2025 Aug 20;36(10):ar124. doi: 10.1091/mbc.E25-04-0154 (PMC12483326; doi:10.1091/mbc.E25-04-0154)
Supplement: Supplementary file 1 [file mbc-36-ar124-s001.pdf]

Supplemental Materials

Molecular Biology of the Cell

Islam *et al*.

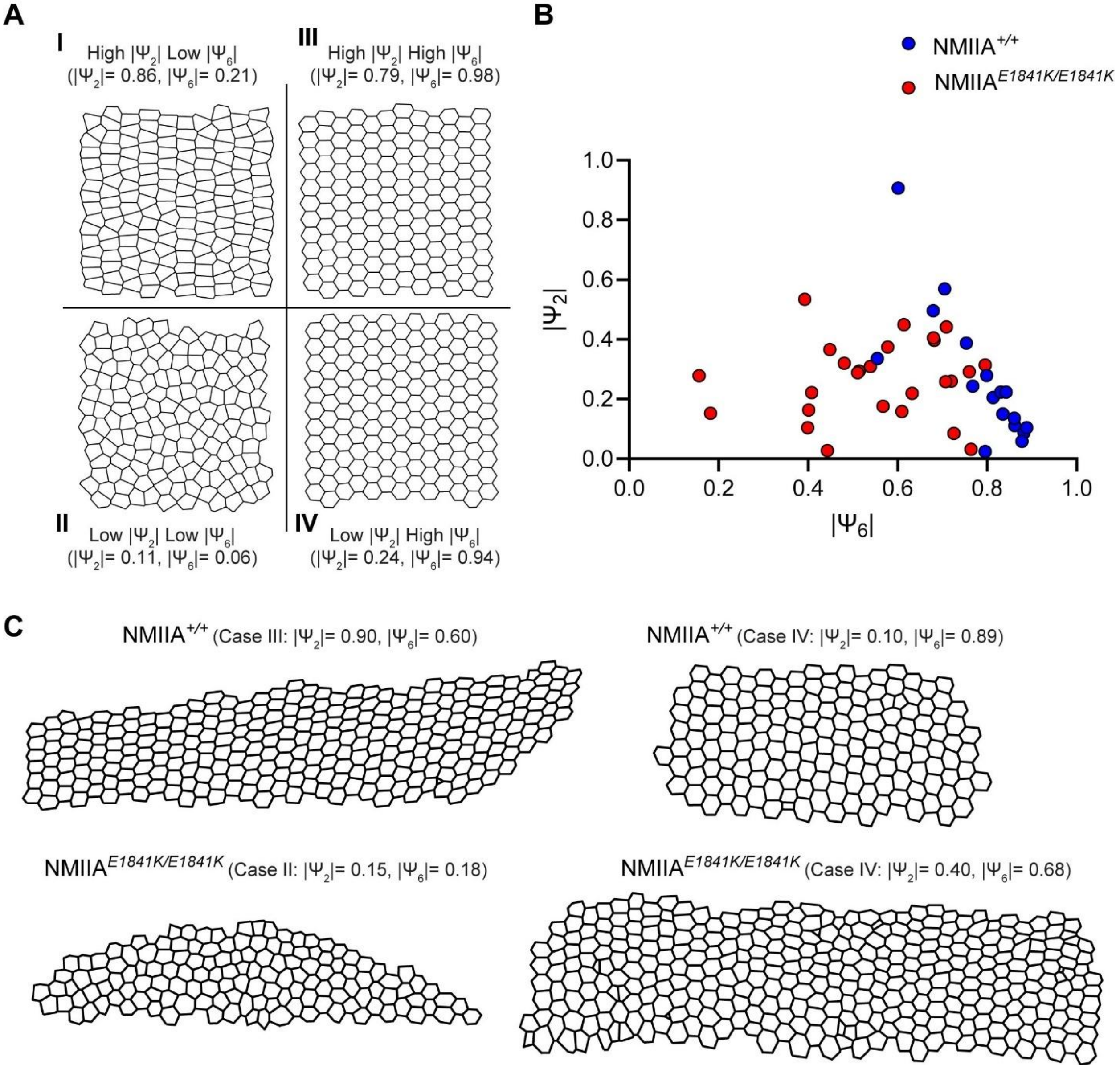

Supplementary Figure 1. Altered packing geometry in NMIIA$^{E1841K/E1841K}$ MR cells. (A) Models of different types of organization are shown based on nematic order |Ψ2| and hexatic order |Ψ6| values. (B) The nematic order |Ψ2| and hexatic order |Ψ6| for NMIIA$^{+/+}$ and NMIIA$^{E1841K/E1841K}$ lenses. Each dot represents a lens image (N=17-26 lens images). NMIIA$^{+/+}$ lenses normally have low |Ψ2| and high |Ψ6|. NMIIA$^{E1841K/E1841K}$ lenses exhibit two types of organization: low |Ψ2|, high |Ψ6| and low |Ψ2|, low |Ψ6|. (C) Representative examples of different types of organization in NMIIA$^{+/+}$ and NMIIA$^{E1841K/E1841K}$ lenses. NMIIA$^{+/+}$lenses normally exhibit type III and IV (more common) organization, whereas NMIIA$^{E1841K/E1841K}$ lenses typically exhibit type II and IV organization.

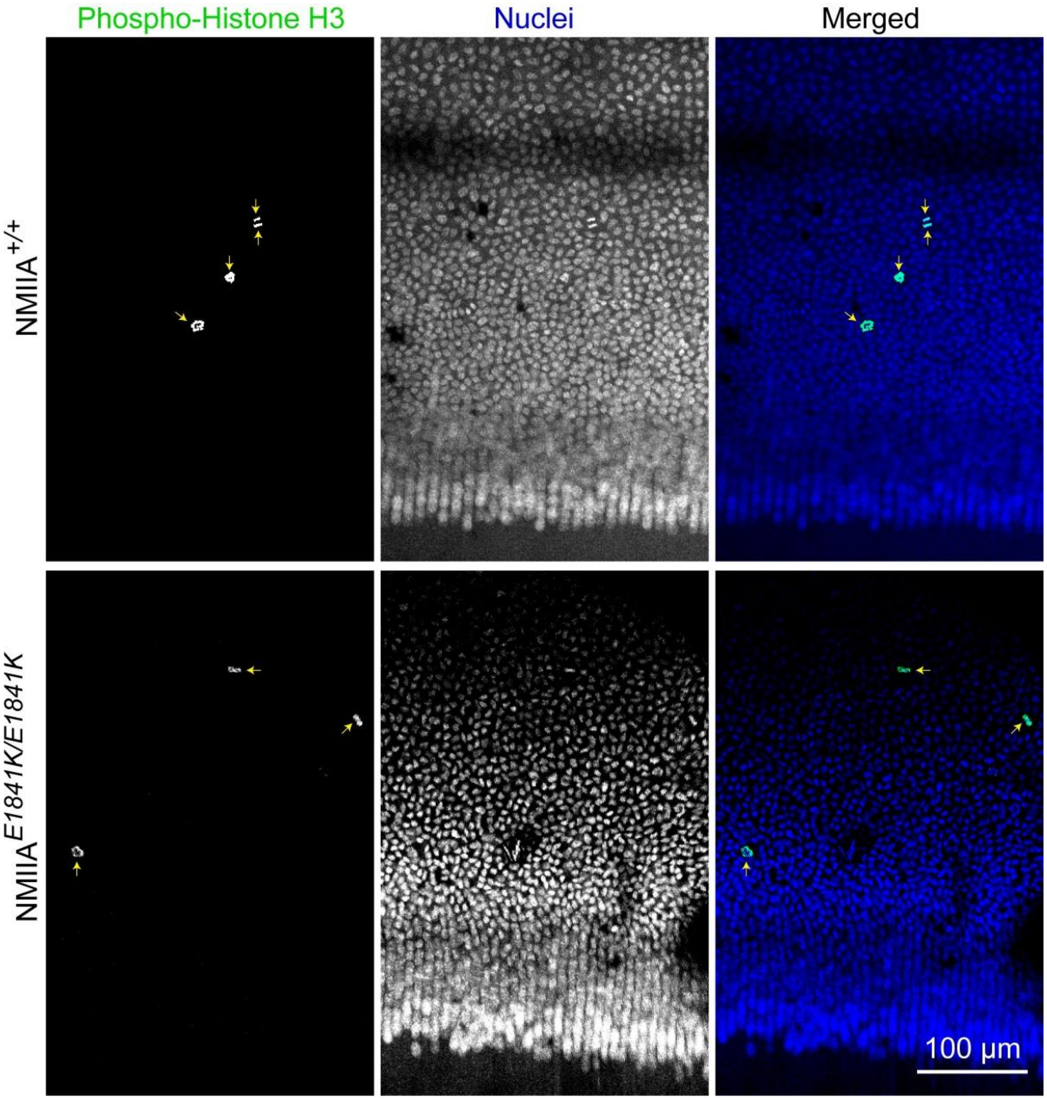

Supplementary Figure 2. The total number of phosphorylated histone-H3 positive cells is unaffected in NMIIA$^{E1841K/E1841K}$ lenses. Formaldehyde fixed whole lens cells labeled for nuclei (blue) and phosphorylated histone H3 at ser10 (green) (mitotic marker). Phospho-Histone H3 positive nuclei (indicated by yellow arrows) are usually observed somewhat anterior to the MR cells at the equator. Scale bar, 100 µm.

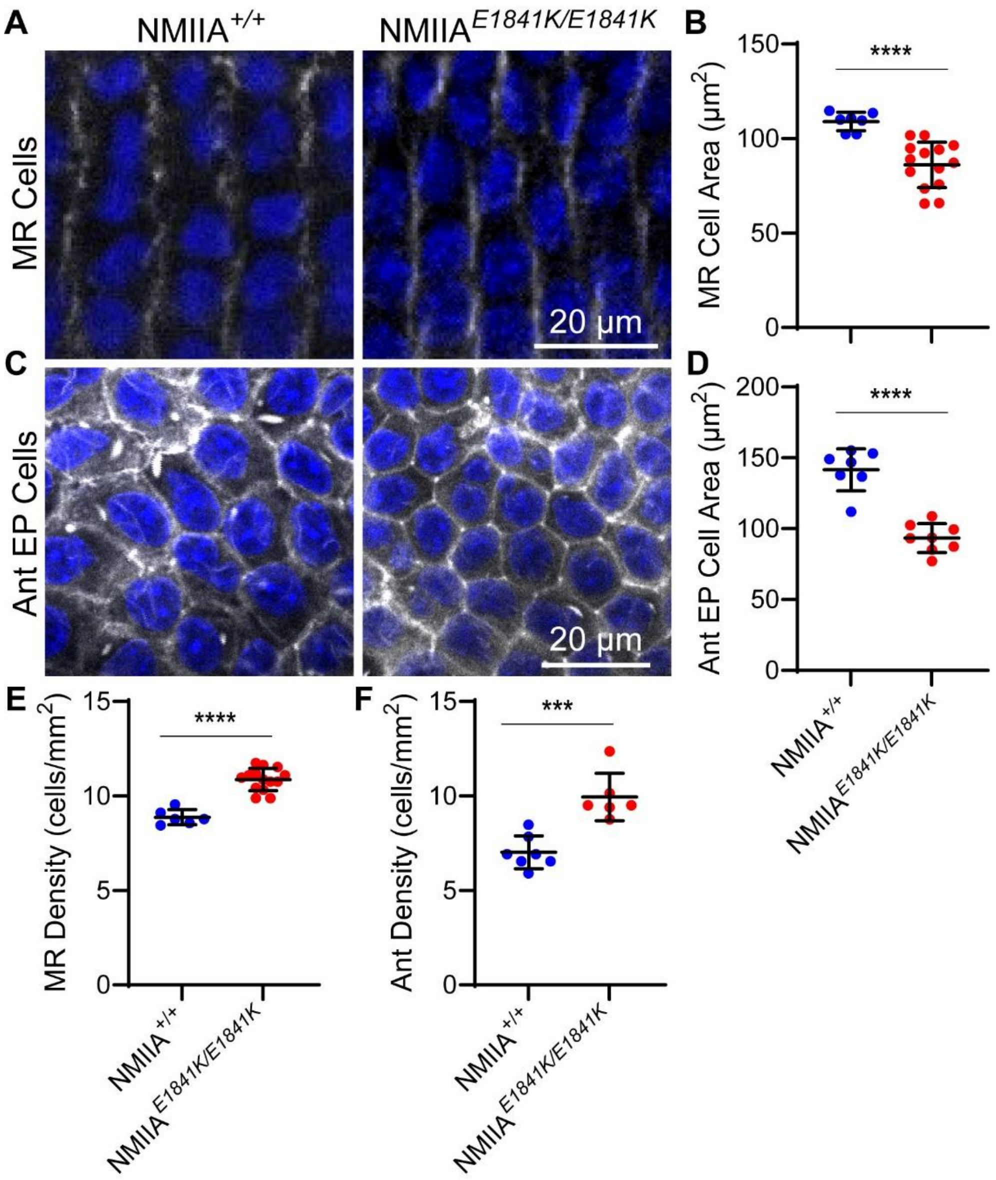

Supplementary Figure 3. Lens MR and anterior epithelial cells are smaller in the NMIIA$^{E1841K/E1841K}$ lenses. Formaldehyde-fixed whole lens cells labeled for nuclei (blue) and rhodamine-phalloidin for F-actin (grey). (A) Representative images at XY plane of lens MR cells. At the mid-lateral domain of MR cells, F-actin is enriched at the AP (vertical) edges. The F-actin labeling shows more rows of MR cells in the NMIIA$^{E1841K/E1841K}$ lenses. There are also more nuclei in the NMIIA$^{E1841K/E1841K}$ lenses compared to the NMIIA$^{+/+}$ lenses. Scale bar, 20 µm. (B) The average cell area reveals that MR cells are smaller in the NMIIA$^{E1841K/E1841K}$ lenses. Plots reflect mean ± SD. Each dot represents a lens image (N = 7-14 MR images from at least 6 mice per genotype). (C) Representative images at XY plane of lens anterior epithelial (Ant EP) cells. The nuclei labeling shows that there are more epithelial cells in the NMIIA$^{E1841K/E1841K}$ lenses. Scale bar, 20 µm. (D) The average cell area reveals that anterior epithelial cells are smaller in the NMIIA$^{E1841K/E1841K}$ lenses. Plots reflect mean ± SD. Each dot represents a lens image (n = 8-9 images from at least 5 mice per genotype). (E, F) MR and anterior epithelial cell density show higher density in the NMIIA$^{E1841K/E1841K}$ lenses. (E) Plot reflects mean ± SD, where each dot represents a lens image (n = 6-14 images from at least 6 mice per genotype). (F) Plot reflects mean ± SD, where each dot represents a lens image (n = 6-7 images from at least 5 mice per genotype). ***$p<0.001$; ****$p<0.0001$.

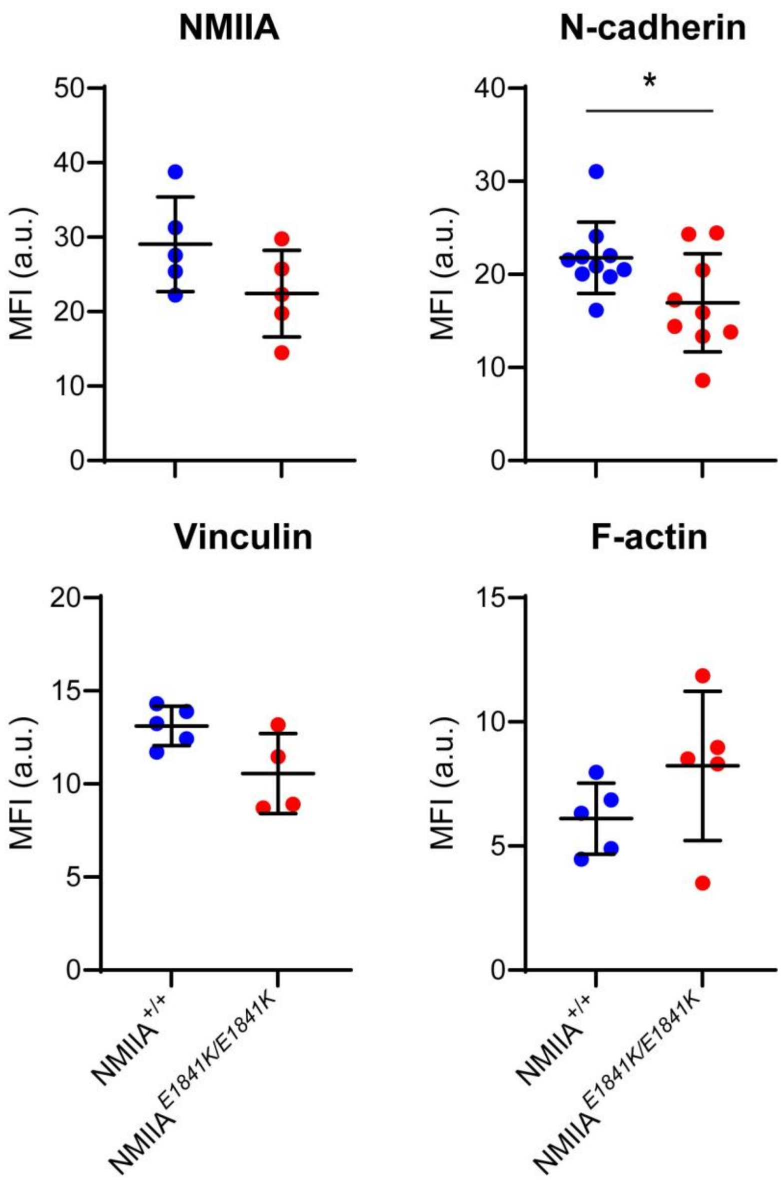

Supplementary Figure 4. The average mean fluorescence intensity (MFI) of N-cadherin, but not NMIIA, vinculin, or F-actin, is reduced at the edges of MR cells from NMIIA$^{E1841K/E1841K}$ lenses. Plot reflects the mean ± SD normalized MFI of NMIIA, N-cadherin, vinculin, and F-actin at MR cell edges. Each dot represents the average per lens image (n=4-9 lenses from at least 3 mice per genotype). *p<0.05.

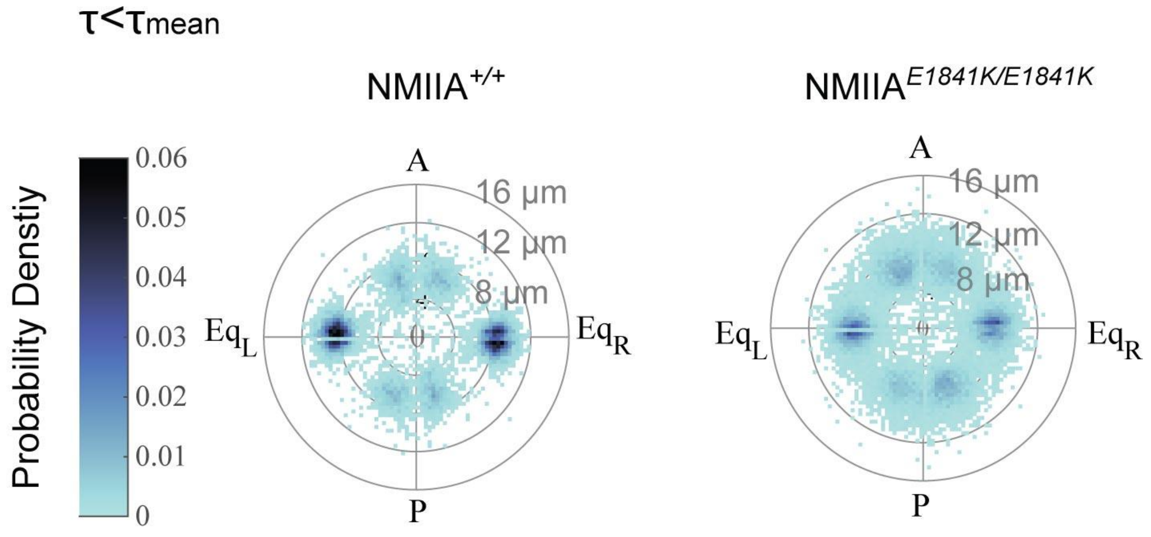

Supplementary Figure 5. The polar distribution of edges with lower-than-mean tensions. In NMIIA$^{+/+}$ cells, low-tension edges are highly polarized along the Eq axis. In contrast, low-tension edges in NMIIA$^{E1841K/E1841K}$ tissues display a six-fold hexagonal symmetry, suggesting that tension is not polarized in NMIIA$^{E1841K/E1841K}$ MR cells.

	NMIIA$^{+/+}$		NMIIA$^{E1841K/E1841K}$	
**Number of Adjacent Cells**	**Total Frequency (%)**	**Average Frequency (%)**	**Total Frequency (%)**	**Average Frequency (%)**
**3**	0.12% (4 cells)	0.14 ± 0.3	0.43% (24 cells)	0.5 ± 0.9
**4**	0.61% (21 cells)	0.60 ± 0.7	1.26% (71 cells)	1.2 ± 0.9
**5**	3.21% (110 cells)	3.1 ± 3.9	8% (451 cells)	8.5 ± 3.8
**6**	92.13% (3161 cells)	92.3 ± 8.0	80.47% (4531 cells)	79.6 ± 7.4
**7**	3.79% (130 cells)	3.8 ± 3.5	9.27% (522 cells)	9.6 ± 3.8
**8**	0.12% (4 cells)	0.1 ± 0.3	0.57% (32 cells)	0.5 ± 0.6
**9**	0.03% (1 cell)	0.02 ± 0.1	0 % (0 cell)	0 ± 0
**Total Number of cells examined**	3431 Cells		5631 Cells	

Supplementary Table 1. % of MR cells with different numbers of adjacent cells. Both total frequency and average frequency (mean ± SD) are reported for both genotypes.

**Materials/Reagents**	**Manufacturer**	**Catalog/Item #**
Methanol	Fisher Scientific	A454-4
Bovine Serum Albumin (BSA) Fraction V >98% Purity	Genesee Scientific	25-529
1X phosphate-buffered saline (PBS)	Thermo Fisher Scientific	14190
Triton™ X-100	Thermo Fisher Scientific	28314
Goat Serum	Invitrogen	A11008
Anti-NMIIA rabbit polyclonal primary antibody (raised against C-terminus)	BioLegend	909801
Anti-N-cadherin mouse monoclonal antibody	Thermo	33-3900
Anti-N-cadherin rabbit monoclonal antibody	Cell signaling	13116T
Anti-Vinculin mouse monoclonal antibody	Sigma	V4505
Paraformaldehyde (16%)	Electron Microscopy Sciences	15710
Agarose	Genesee Scientific	20-101
Rhodamine-phalloidin	Thermo Fisher Scientific	R415
FluoroDish cell culture dishes	World Precision Instrument	FD35-100
CrystalCruz® Adhesive Micro Slides	CrystalCruz	sc-363560
Hoechst 33342	Thermo Fisher Scientific	H3570
Alexa-Fluor-647-conjugated goat anti-rabbit-IgG	Thermo Fisher Scientific	A-21245
Alexa-Fluor-568-conjugated goat anti-mouse-IgG	Thermo Fisher Scientific	A-11031
ProLong® Gold antifade reagent	Thermo Fisher Scientific	P36934
Anti-Ki67 rabbit monoclonal antibody	Cell Signaling	9129S
Feather Double Edge Safety Razor Blades	Amazon	B001G5FOLI
48-Well Cell Culture Plates Flat Bottom Wells	Genesee Scientific	25-108
Vannas-Tübingen Spring Scissors (for dissection)	Fine Science Tools	15003-08
Dumont #5 - Mirror Finish Forceps # 5 (for dissection)	Fine Science Tools	11251-23

Supplementary Table 2. List of materials.
